# Fabrication and characterization of microfluidic devices based on boron-modified epoxy resin using CO_2_ laser ablation for bio-analytical applications

**DOI:** 10.1038/s41598-023-39054-0

**Published:** 2023-08-03

**Authors:** Heba Mansour, Emad A. Soliman, Ahmed M. Fath El-Bab, Yoshihisa Matsushita, Ahmed L. Abdel-Mawgood

**Affiliations:** 1 Department of Polymeric Materials Research, Advanced Technology and New Materials Research Institute (ATNMRI), City of Scientific Research and Technological Applications (SRTA-City), New Borg El-Arab City, 21934 Alexandria Egypt; 2 Mechatronics and Robotics Department, School of Innovative Design Engineering, Egypt-Japan University of Science and Technology (E-JUST), New Borg El-Arab City, 21934 Alexandria Egypt; 3 Egypt-Japan University of Science and Technology (E-JUST), New Borg El-Arab City, 21934 Egypt; 4 Biotechnology Program, Basic and Applied Sciences (BAS) Institute, Egypt-Japan University of Science and Technology (E-JUST), New Borg El-Arab City, 21934 Alexandria Egypt

**Keywords:** Mechanical engineering, Polymer chemistry

## Abstract

CO_2_ laser ablation is a rapid and precise technique for machining microfluidic devices. And also, low-cost epoxy resin (ER) proved the great feasibility of fabricating these devices using the CO_2_ laser ablation technique in our previous studies. However, such a technique has shown negative impacts on such ER-based microfluidics as rough surface microchannels, and thermal defects. Therefore, incorporating different proportions of boric acid (BA) into epoxy resin formulation was proposed to obviate the genesis of these drawbacks in ER-based microfluidics. The structural and optical properties of plain ER- and B-doped ER-based chips were characterized by Fourier transform infrared (FT-IR) and UV/Vis spectral analyses. Furthermore, their thermal properties were studied by thermo-gravimetric (TGA) and differential scanning calorimetric (DSC) analysis. A CO_2_ laser ablation machine was used in vector mode to draw the designed micro-channel pattern onto plain ER- and B-doped ER-based chips. The quality of microchannels engraved onto these chips was assessed using 3D laser microscopy. This microscopic examination showed a noticeable reduction in the surface roughness and negligible bulge heights in the laser-ablated micro-channels. On the other hand, overall and specific migration using gravimetric methods and gas chromatography-mass spectrometry (GC–MS), respectively, and PCR compatibility test were performed to explore the convenience of these micro-plates for the biological reactions. These findings validated the applicability of B-doped ER-based microfluidics in bio-analytical applications as a result of the effective role of boric acid in enhancing the thermal properties of these chips leading to get micro-channels with higher quality with no effect on the biological reactions.

## Introduction

In the last ten years, polymers have replaced silicon/glass as alternate substrates for microfluidic devices because of the versatility in their chemical, optical, and physical properties^1,2^. Moreover, they are often considered lower costly compared with silicon/glass and easy to process with several fabrication methods. Currently, both thermoplastics and thermosets e.g., PDMS (polydimethylsiloxane)^3^, PMMA (polymethylmethacrylate)^4^, PC (polycarbonate)^5^, and PS (polystyrene)^6^ are widely employed in microfluidics.

Epoxy resins are extensively used in industry as construction materials, coatings, adhesives, and matrices for advanced composites because of their superior mechanical and chemical resistance, electrical properties, and resistance to moisture^7^. Epoxy resin readily available in the marketplace has several crucial characteristics that make it a strong candidate for use in microfluidics, including a high young's modulus, high optical transparency, autoclavability, hydrophobicity, negligible migration, ease of handling, and low cost^8^. Concurrently, epoxy polymers exhibit low thermal stability and high flammability^9^.

Boron compounds are relatively nontoxic, naturally available, ecofriendly and have proven to enhance the fire retardancy of epoxy polymers, wood, cellulose, cotton, etc. Amongst the boron compounds, boric acid is well known for its fire retardancy and exerts its flame retardant properties at a temperature well below the normal pyrolysis temperature of polymeric materials. As an inorganic reagent, boric acid shows relatively low toxicity^10^. In this context, fine powder of boric acid (particles dia. < 40 µm) was previously used as flame-retardant filler for epoxy resin (1–10% of ER mass). The thermal stability of the epoxy composite was increased with increasing amount of boric acid, and this enhancement was attributed to decrease mobility of epoxy phase in the vicinity of the boric acid particles and the more uniform distribution of boric acid in epoxy matrix^11^. On the other side, boron-containing compounds including boric acid have been also used as curing agents^9,12^. Similarly, BA has been utilized as a cross-linking agent for cellulose fibers/polyvinyl alcohol composite^13^, polyvinyl alcohol and ethyl cellulose films as drug delivery systems^14^, and polyvinyl alcohol adhesive^15^. The cross-linking capability for boric acid with such biodegradable hydrophilic polymers (cellulose, cellulose derivatives, and PVA) was explained on the basis of the dispersion uniformity to a coulombic acid/base reaction of the acidic protons of the three –OH groups on this simple triprotic acid (boric acid) with the hydroxyl groups of the polymer molecules. The high reactivity of boric acid can be attributed to the presence of vacant *d*-orbital in trivalent boron atoms, which causes it to react rapidly with various nucleophiles to form complexes^15^. Likewise, monomeric and polymeric epoxy resins may also include reactive -OH groups, which arise from the ring-opening reaction of epoxides, reacting with boric acid to result in an additional cross-linking. As well as being examined as a curing accelerator to the embodiments that contain epoxy resin, curing agent, and catalyst where they cure more rapidly if contain a stoichiometric excess of boric acid compound over the catalyst^16^. Despite the mentioned above benefits of the incorporation of boric acid in an epoxy matrix, it may cause coloration of the microfluidic platforms with pale yellow color declining their transparency and subsequently reducing the quality of the latter^7,17^ because transparency considers one of the most critical quality peculiarities of microfluidic devices^18^.

Otherwise, epoxy resin-based microfluidic devices have already shown good processability using the rapid and cost-effective CO_2_ laser ablation approach in our previous study. However, they have revealed a downside because the laser intensity applied in the micro-patterning led to defects by the action of the resulting heat as bulge heights and surface roughness^8^. Therefore, this work will address enhancing the thermal stability of ER-based microfluidic platforms with doping epoxy resin with boric acid. Regarding the significance of uniformity of the distribution of processing aids in the epoxy resin matrix in achieving the effective role^19,20^ and tuning the act of boric acid to enhance the thermal properties of the microfluidic devices with no negative impact on the other quality merits, before becoming a part of the composition, the boric acid is dissolved in methanol which a reactive diluent for the main component of the compositions (epoxy resin). Such methodology was proposed to enhance the boric acid distribution in the ER matrix and consequently, the thermal stability and micro-channel quality of the boric acid-doped epoxy resin (B-doped ER)-based microfluidic platforms with maintaining their transparency. Furthermore, the optimization of laser micromachining parameters such as laser power (P) and scanning speed (U) was studied. Further, other critical assessments on these boric acid-doped epoxy resin-based microfluidic platforms' are carried out to explore their convenience for biotechnological applications. These assessments involved the measurement of the potential migrants in addition to PCR compatibility testing.

## Materials and methods

Diglycidyl ether of bisphenol-A (DGEBA) with epoxy equivalent weight (EEW) = 185–190, and curing agent (hardener); modified cycloaliphatic polyamine were purchased from Green Build Chemical Company (Egypt). Boric acid, H_3_BO_3_, MW (g/mol) = 61.83 (Carlo Erba Reagents). Methanol HPLC grade MW = 32.04 from Fisher Scientific UK.

### Microfluidic platforms Fabrication

#### ER- based plate fabrication

The fabrication method of pristine ER- and B-doped ER–based chips was taken place by compounding the components and casting technique adapted from the method of Lau and Lu^21^ with some modifications. Briefly, different proportions of boric acid were dissolved in methanol at room temperature to form a clear alcoholic solution of boric acid with various concentrations (5, 10, 15, and 20)% (w/v), as in Table 1. A predetermined amount of boric acid solution (50% w/w of the resin) was added to the resin, and the mixture was sonicated in a Sonic vibrator (500 W). The mixture was placed on a hot plate at 70 °C for 45 min to evaporate the solvent completely, followed by a degassing process in a vacuum for 4 h for all specimens. Finally, the hardener was added to the mixture at epoxy resin: hardener ratio of 2:1.25 (w/w) and homogenized. The epoxy mixture was cast in a silicone mold. Pre-curing step was done on a hot plate at 50 °C for 10 min with stirring and then curing at room temperature for 24 h. Control specimens (0% boric acid) were fabricated for comparison using the same preparation and curing procedures.

**Table 1:** Designed Formulations of epoxy resin systems.

Conc. (%)	Epoxy resin (gm)	Boric acid (gm) in methanol (5 ml)	Hardener (gm)
0	10	0	6.25
5	10	0.25	6.25
10	10	0.5	6.25
15	10	0.75	6.25
20	10	1	6.25

#### Laser micro-machining setup

**Figure 1:**
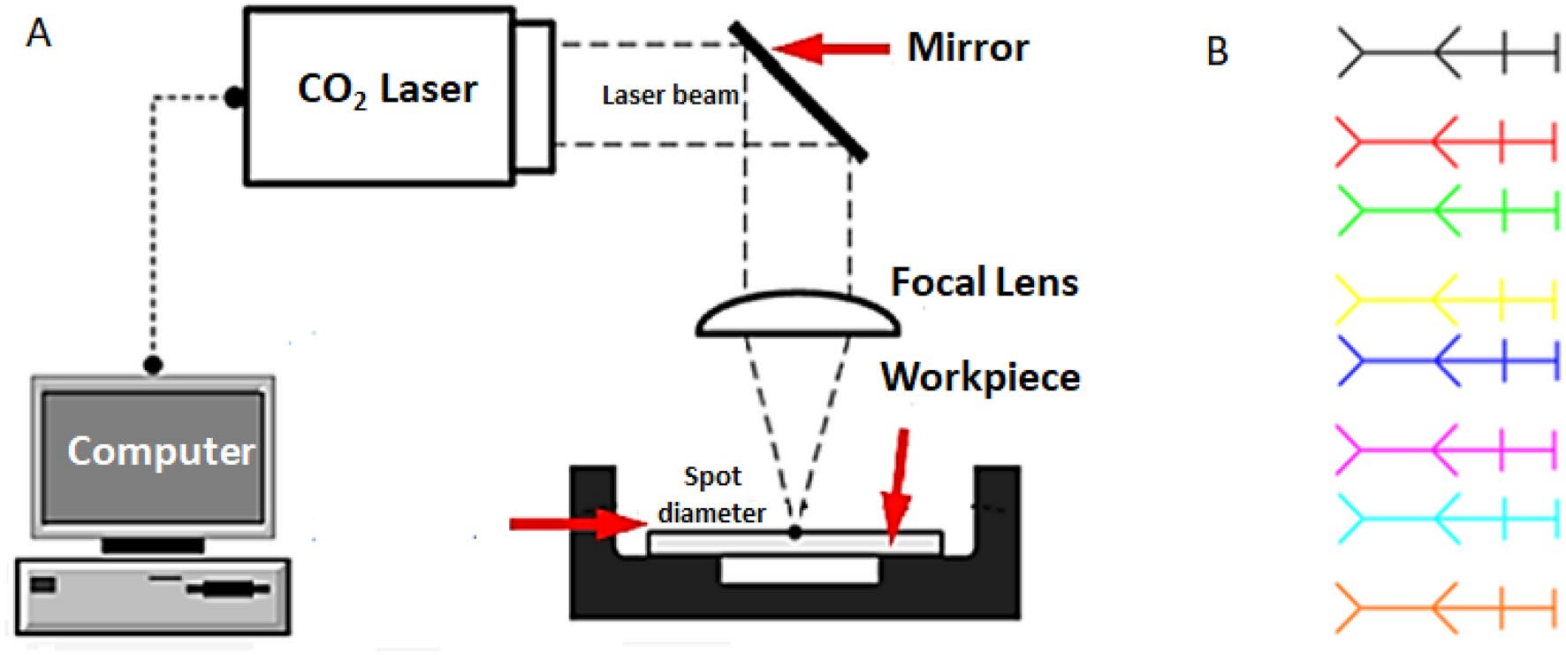
Schematic representation of CO_2_ laser ablation process (**a**) and eight-line design (**b**).

CO_2_ laser micro-machining is an efficient and cost-effective method of producing a wide range of polymeric microfluidic devices^22^. The pre-fabricated epoxy resin cast chips (3 mm thick) were micro-machined using a commercial bench top CO_2_ laser (Universal Laser System, VLS 3.5, USA) with a maximum power of 30W (Fig. 1a). The micro-channel pattern was designed by using CorelDraw©X5 2010 software, a computer-aided design (CAD) tool, associated with the laser cutter. Vector mode was adopted for cutting lines widths below 200 μm. The design was plotted on eight lines with different colors at a time due to the ability of the plotter to accommodate eight different laser settings in one pass (Fig. 1b). Varying laser settings were employed to optimize the micro-patterning process where average power (P) ranged from 1.8 to 3.6W and scanning speed (S) ranged from 5 to 20 mm/s. The quality characteristics for the resulting micro-channels include the width, depth, and surface roughness (Ra) of the micro-channels and bulge heights was assessed using 3D laser microscope (Keyence VK- × 100). Each experiment was conducted three times, and then the average and standard deviation were calculated.

### Consent to participate

Informed consent was obtained from all authors who participated in the study.

## Microfluidic substrate and platforms characterization

### Fourier transform infrared (FT-IR) spectral analysis

The chemical structures of cured epoxy resin and embodiments prepared with the incorporation of different concentrations of boric acid were studied by Fourier transform infrared (FT-IR) spectral analysis. FTIR spectra were recorded for KBr discs on a Shimadzu FTIR-8400S spectrometer in a range of 400–4000 cm^−1^ at a resolution of 4 cm^−1^ and 32 scans.

### Transparency measurement

The epoxy resin shows a transparent aspect in its pure state. To explore the impact of the incorporation of boric acid in the epoxy resin compositions on the optical properties of microfluidic plate, the transparency of the epoxy resin specimens was measured using UV/Vis spectrophotometer (Hitachi Model U-3900).

### Hardness measurement

Vickers hardness of epoxy resin plates was carried out using MICRO Hardness Tester Shimadzu (HMV-2 series), where test force (490.3 mN) which is equal to 0.0499 kgf load, was applied for 10 s. The hardness value HV was calculated from the indentation load and the diagonal of the Vickers imprint.

### Thermogravimetric analysis (TGA)

The thermal stability of the epoxy resin specimens was assessed using Shimadzu TGA-50 thermogravimetric analyzer by heating the specimen from ambient temperature to 800 °C at a rate of 10 °C/min under a nitrogen atmosphere.

### Differential scanning calorimetry (DSC)

The thermal transition in epoxy resin specimens were studied by differential scanning calorimetry (DSC) using a Discovery X3 DSC (TA Instruments, USA). The thermal behavior was studied under non-isothermal linear heating (10 °C /min) in a nitrogen atmosphere from room temperature to 800 °C.

### Differential thermal analysis (DTA)

Differential thermal analysis (DTA) was performed using a Discovery DTA (TA Instruments, USA). The analysis was performed at a heating rate of 10 °C min^−1^ starting from room temperature to 800 °C with nitrogen purge gas.

### Autoclaving endurance

As well-known, all substrates and devices in biological applications which in contact with biological components, organs, or cells have to be sterilizable because their contamination could damage these biological systems and affect the accuracy and reproducibility of such analyses. Autoclaving is the most widely technique used for sterilization because of its efficiency and low cost. The capability of epoxy resin-based plates to withstand autoclaving was assessed by conducting a standard autoclaving with saturated steam at 121 °C under the pressure of approximately 15 pounds per square inch for 20 min using MaXterile 60 autoclave^23^. The autoclaving standability was evaluated by measuring their dimensions and weight accurately before and after autoclaving. For the pristine cured epoxy resin-based chips, it was found to withstand autoclaving as indicated in our previous study^8^. Herein, the autoclaving standability of the B-doped ER-based chips was assessed to explore the impact of the incorporation of boron compounds in the epoxy resin matrix.

### Overall and specific migration testing

To guarantee the aptness of epoxy resin-based microfluidic devices for bio-analytical application, the non-presence of any substances that could interfere with the biological components is highly necessary for these analyses to ensure their accuracy and sensitivity. The potential release of the components from B-doped ER compositions was measured according to the method reported in a study by Cardama and his colleagues^24^. Concisely, a B-doped ER specimen was immersed in distilled water as a polar simulant or in dichloromethane as a low polar simulant and heated with shaking at 100 °C for 2 h. An aliquot of the aqueous simulant (2 ml) was then heated to evaporate the simulant and measure the overall migration by weighing the remaining solid (all migrants) by using the sensitive balance. For measuring the specific migration, these solid residues were dissolved in methanol (2 ml), and the resulting methanolic solution was qualitatively analyzed by gas chromatography coupled with a mass spectrometer (Shimadzu GC-MS-QP2010).

### Polymerase chain reaction (PCR) compatibility testing

This test is performed to examine the effect of contacting a polymerase chain reaction (PCR) mixture on cured epoxy resin plates.

#### Polymerase chain reaction

The polymerase chain reaction (PCR) includes primer-mediated enzymatic amplification of DNA. This technique uses the ability of DNA polymerase to synthesize a new strand of DNA complementary to the template strand. PCR Master Mix (2X) is a pre-mixed solution containing Dream Taq™ DNA polymerase, MgCl_2_, optimized Dream Taq™ buffer, and dNTPs that save time and decrease contamination. It can cause robust amplification of genomic DNA (up to 6 kb). The entire length of the EGFR exon 19 gene was amplified using the oligonucleotide primers Ex19 Fw (5′-AGCATGTGGCACCATCTCAC-3′) and Ex19 Rv (5′-ATGAGAAAAGGTGGGCCTGA-3′). The reaction mixtures volume was 25 µl contained 0.1 ng of sample DNA, 12.5 µl 2 × PCR Fast Gene Taq 2 × Ready Mix (Nippon Genetics, Germany), a 200 nM concentration of both primers, and 2.5 µl bovine serum albumin (BSA). BSA weight (1 µg/µl) dissolved in freshly prepared phosphate buffer saline (PBS) solution (pH 7.4)^25^. PCR cycling was performed in a Lab cycler (SensoQuest GmbH, Germany) with the following parameters: one cycle of 4 min at 94 °C, followed by 35 cycles of denaturation for 1 min at 94 °C, annealing for 30 s at 58 °C, extension for 2 min at 72 °C and final extension at 72 °C for 5 minutes^26^. The reaction mixture was added to a PCR tube containing a piece of ER and ER coated with BSA to test its effect on the PCR test efficiency.

#### Gel electrophoresis

Nucleic acid bands (PCR products) were identified and visualized by running in 1.0% agarose gel in 0.5 × TBE buffer system with 90 V for 35 min. in horizontal gel electrophoresis apparatus (Cleaver Scientific, UK), followed by staining with ethidium bromide, a non-radioactive marker, and pictured on UV Trans illuminator, (Nippon Genetics, Germany). Radiolabeled DNA fragment sizes were approximated using eco in Action (ready-to-use) 100 bp DNA Ladder (H3 RTU).

## Results and discussion

### Characterization of ER and B-doped ER

#### FTIR spectra

**Figure 2:**
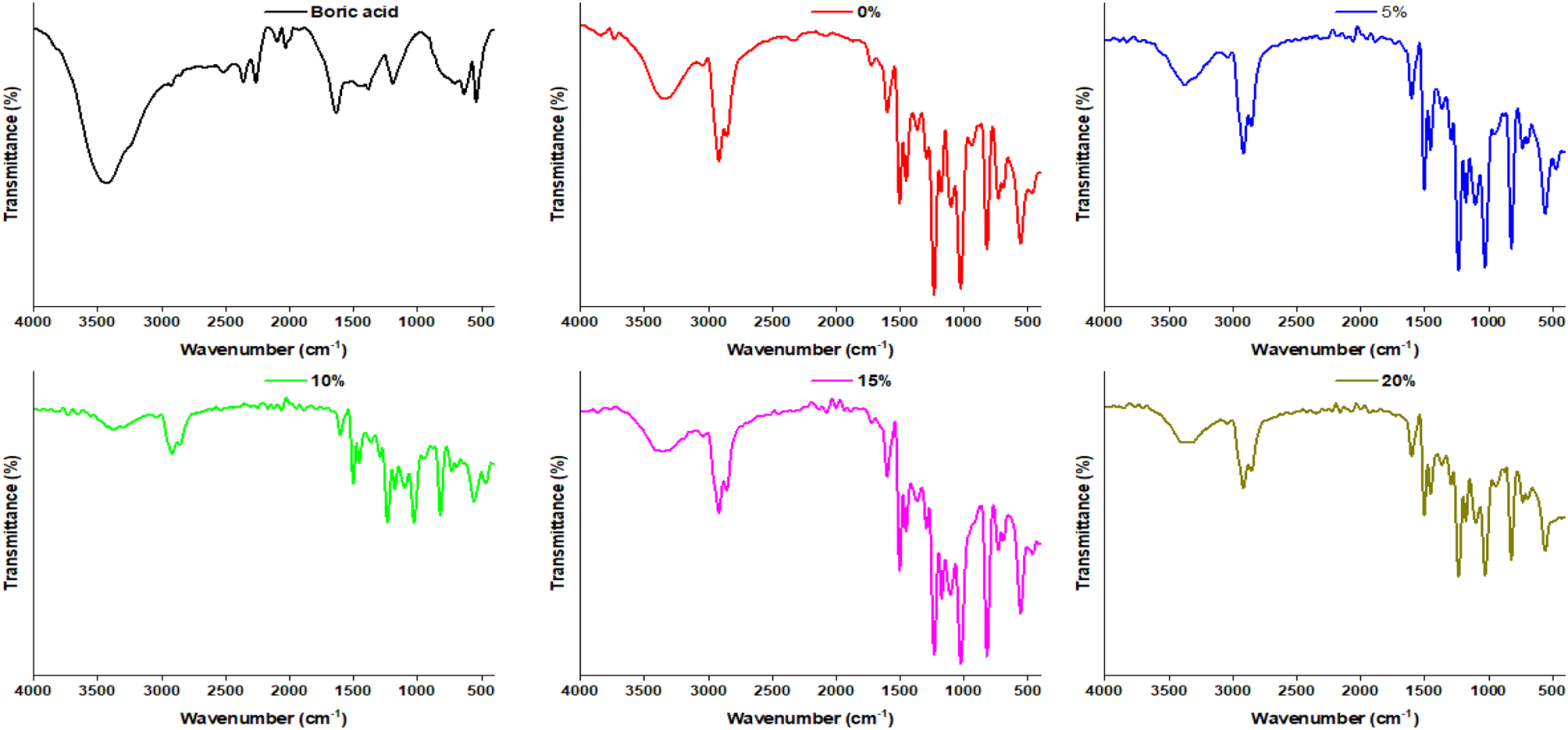
FT-IR spectra of plain epoxy resin and boron-doped epoxy resin prepared with different concentrations of boric acid (5, 10, 15, and 20%).

FTIR is an analytical technique used to identify organic, polymeric, and, in some cases, inorganic materials. Each molecule or chemical structure will produce a unique spectral fingerprint, making FTIR analysis a great tool for chemical identification. FTIR spectra of boric acid, pristine ER, and ER doped with boron by incorporating varied proportions of boric acid in epoxy resin matrix were shown in Fig. 2. The FTIR spectrum of boric acid as a component of B-doped ER composition revealed a sharp band at 541 cm^−1^ corresponding to the bending vibration of B–O–B. The band at 636 cm^−1^ is ascribed to the deformation vibration of B–O. The band at 1192 cm^−1^ is attributed to the in-plane bending vibrations of B–O–H. The band observed at 2263 cm^−1^ could be caused by stretching vibrations of atoms in the B–O bonds. The stretching vibrations of O–H are supported by the broad band at 3461 cm^−1^. The absorption band at 1385 cm^−1^ is belonging to asymmetric stretching vibrations of B–O^27,28^.

The prepared cured epoxy system was characterized by IR for to confirm its composition and crosslinking of polymeric epoxy resin (Fig. 2). The cured ER spectrum displayed a broad doublet absorption band at 3394 cm^−1^ is due to overlapping the adsorption peaks belonging to the –NH_2_ vibration of amine compounds and stretching vibration of OH of epoxy resin. Adsorption peaks at 1605, 1580, 1510, 1455 cm^−1^ are assigned for Ar–C=C–H stretching vibrations. The absorption bands due the gem-dimethyl C (CH_3_)_2_ appeared at 1384 and 1360 cm^−1^ is. The two bands at 729 and 693 cm^−1^ may be attributed to out of plan bending of aromatic rings. The peak at 1182 cm^−1^ is due to C–O stretching of aromatic ring of DGEBA. The aliphatic –CH_2_ and –CH_3_ vibrations are seen in the region 3000- 2850 cm^−1^ for both the polyamine adducts. The absorption peaks appeared at 1036 and 1240 cm^−1^ is due to symmetric and asymmetric stretching vibrations of aromatic ether C–O–C). The disappearance of the peaks at 3056 and 915 cm^−1^ belonging to vibration of C–H and C–O of epoxy group, respectively, in addition to the appearance of the characteristic band due to C–N stretching vibrations at 1108 cm^−1^ indicating the opening of epoxy rings and confirming the curing process^29^.

The spectra of ER containing boron additives exhibited characteristic broad absorption peaks at 3340 (O–H and N–H stretching vibrations), bimodal peaks at 2875 and 2931 cm^−1^ (aliphatic C–H symmetric and asymmetric stretching vibrations), 1607 and 1510 cm^−1^ (aromatic C=C stretching vibrations). The adsorption peak replicated at 1030 cm^−1^ with medium intensity as a result of overlapping the frequencies of C–O–C stretching with the corresponding due to B–O–C groups, which confirms the reaction between boric acid and epoxy resin^30^. However, this peak is 1030 cm^−1^), which already exists in the plain epoxy resin^31^. Also, absorption peaks with medium intensity occur at 1330–1375 cm^−1^ due to the stretching vibration of the B–O and B–N bonds.

In comparison with neat epoxy resin, the stretching vibration of O–H in the modified epoxy resin shifted to lower wavenumber region, in addition to a broadening of the hydroxyl group is attributed to the cross-linking reaction and formation of hydrogen bonds originated from reaction of the hydroxyl groups of cured epoxy resin and boron containing species. Moreover, the presence of systematic increases in boric acid concentrations resulted in increased intensity in frequencies associated with B–O and B–N stretching and the characteristic band due to stretching vibrations of C–N bond (at 1108 cm^−1^) was observed with increasing the proportion of boric acid incorporated in the epoxy resin. These findings prove the reaction of the boron-containing species with the cured polymeric epoxy.

#### Optical transmittance

**Figure 3:**
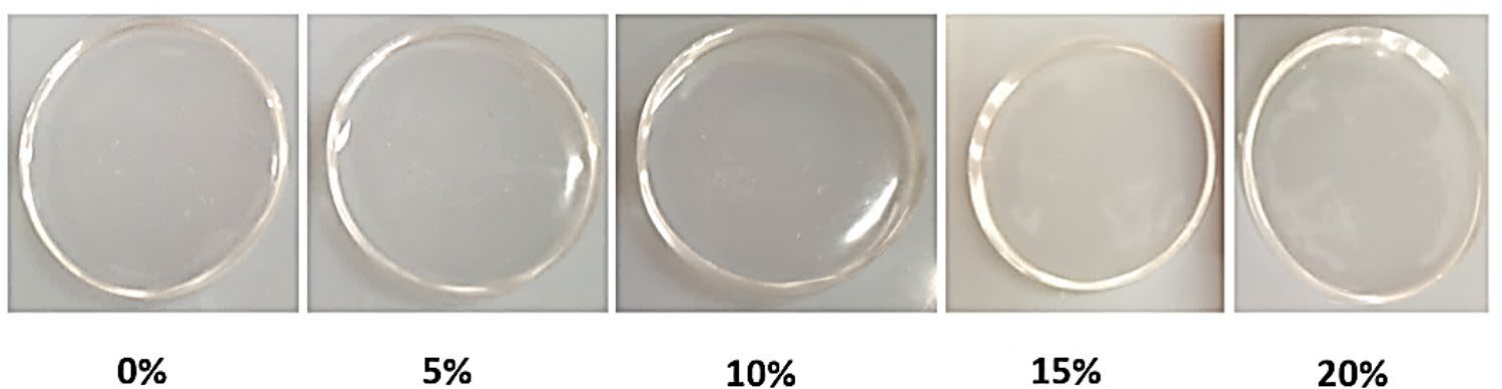
Appearance of the pristine ER- and B-doped ER-based cast discs prepared with varying proportions of boric acid.

Optical transmittance (transparency) is an essential characteristic of microfluidic devices, and it should exceed 90% to ensure good visability^32^. The optical transmittance values of plain ER-based and B-doped ER cast discs are listed in Table 2 and their appearance are shown in Fig. 3. These results indicated that the transparency of plain ER specimen is higher than those for B-doped ER specimens. Further, the boron-modified epoxy resin specimens exhibited a noticeable reduction in transparency with increasing the proportion of boric acid in their compositions. Notwithstanding, all fabricated B-doped ER specimens still had transparency values (> 96%) suitable for microfluidic platform applications. On the contrary, in a previous study, the incorporation of a micro-sized powder of boric acid as a flame-retardant filler to epoxy resin led to the loss of the transparency of epoxy resin and its coloration with dark yellow^33^. This variation can be explained on the basis that the compounding method provided in this study by dissolving boric acid in methanol before incorporation into an epoxy resin matrix led to the formation of new boron-containing compounds in addition to better distribution uniformity for these compounds in the epoxy resin matrix and which would produce boron-modified epoxy resin having better optical properties than those for BA/ER composites prepared in the study mentioned before.

**Table 2:** Transparency and hardness of plain ER and B-doped ER prepared with varying boric acid concentrations.

Boric acid (%)	0	5	10	15	20
Transparency (%)	99.6	98.4	98.0	98.2	96.1
Hardness (HV.)	12.4	16.7	30.5	35.5	41.4

#### Hardness

Hardness is well-defined as a material's ability to resist penetration, indentation, and abrasion. Vickers hardness values of plain ER and B-doped ER specimens were tabulated in Table 2. These data exhibited that the hardness of B-doped ER specimens was increased with increasing BA proportion used in the epoxy resin compositions. The B-doped ER specimen fabricated using 20% boric acid showed the highest hardness, which was higher than that of the plain epoxy resin by about 30%. Conversely, it has been previously reported in another study that an incorporation of boric acid powder leads to lower the hardness of the boric acid-loaded ER specimens. This difference can be due to the weak interfacial interactions between the epoxy polymer and boric acid particulates in the produced composite and heterogeneity of filler dispersion causing voids formation in the resin matrix, particularly at higher filler contents7. But, increasing the hardness for the boron-modified epoxy resin specimens prepared in this study can be referred to as enhancing crosslinking density in the epoxy resin matrix, which confines the motion of polymer chains and consequently increases hardness^34,35^.

#### TGA thermograms

**Figure 4:**
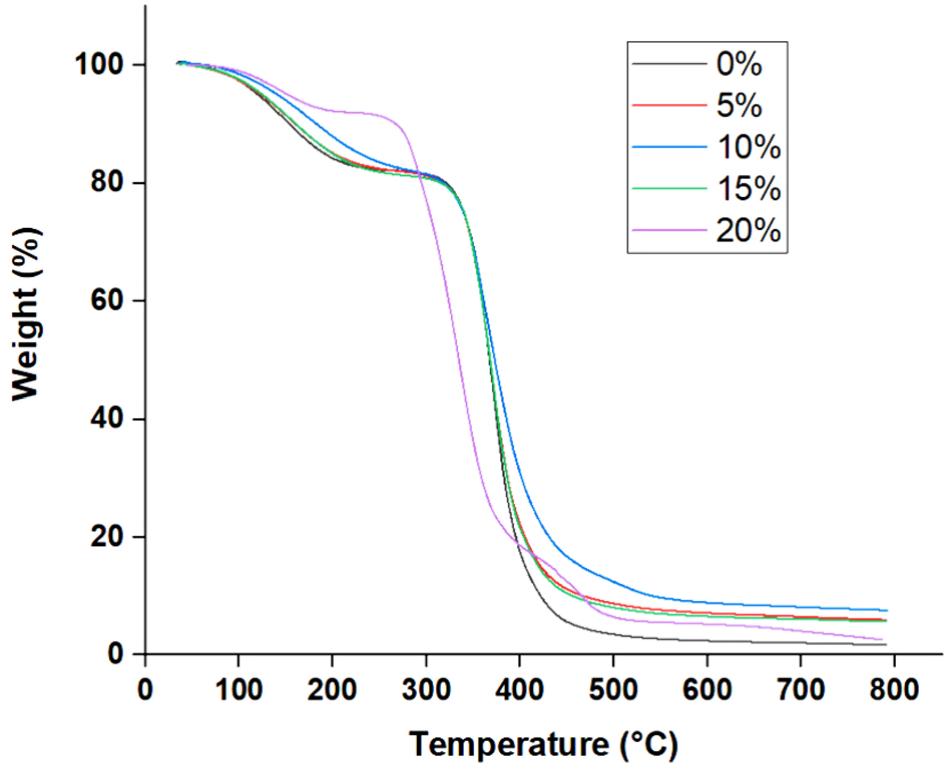
TGA thermograms of plain ER and the B-doped ER specimens prepared at varying boric acid concentrations.

Considering the thermal effect of the laser ablation procedure that may raise the treated substrate temperature to 150 °C maximum, as mentioned in several previous studies^36,37^, this study has aimed to enhance the thermal stability of ER substrate via compounding of epoxy resin with boric acid (flame retardant) into the resin matrix. Therefore, thermo-gravimetric analysis (TGA) has been used to assess the thermal stability of ER and B-doped ER specimens. TGA thermograms depicted the relationship between the weight losses owing to the thermal decomposition of specimens with temperature as shown in Fig. 4. The temperature of degradation at which the weight loss is 5, 10, 20, 50, 70, and 90% from the initial weight of ER specimens has denoted with *T*_5_,* T*_10_,* T*_20_, *T*_50_, *T*_70_, and *T*_90_, respectively. The thermo-oxidative degradation of the plain epoxy resin was nearly completed at 790 °C, and the mass residue was ~ 1.75%. Therefore, the residues of B-doped ER specimens at 790 °C were used to evaluate their thermal stability. The data obtained from the TGA thermograms of the prepared epoxy resin compositions are given in Table 3.

**Table 3:** Degradation temperatures of the ER and B-doped ER specimens prepared at varying boric acid concentrations.

Boric acid (%)	Degradation temperatures (°C)					
	*T*_5_	*T*_10_	*T*_50_	*T*_70_	*T*_90_	Residue at 790 °C (%)
0	120.45	154.05	367.41	382.89	422.06	1.75
5	124.24	160.38	368.08	386.72	468.55	6.02
10	131.05	173.51	369.45	395.58	538.83	7.63
15	125.44	161.14	368.11	386.23	456.81	5.69
20	152.42	267.79	334.64	358.83	465.99	2.63

These findings exhibited that incorporating the boric acid in the epoxy resin compositions has an efficient role in the enhancement of the thermal stability of the boron-doped epoxy resin casted specimen. The degradation temperatures for such boron-modified epoxy resin specimens were higher than their counterpart of plain epoxy resin specimens. Increasing the proportions of boric acid employed in the fabrication process led to elevating.

The degradation temperatures. *T*_*5*_ and *T*_*10*_ of B-doped ER cast specimens prepared using 20% of BA was ~ 152 and ~ 268 °C, respectively, and higher than those corresponding for plain epoxy resin by 32 and 114 °C, respectively. The residue of the B-doped ER cast specimen (10% BA) at 800 °C is 7.5%, while the neat epoxy polymer almost decomposes completely.

The incorporation of boric acid (H_3_BO_3_) into epoxy resin leads to a significant modification of the structure and properties of epoxy thermosets with increasing thermal stability of the modified resin. However, the compounding methodology applied in this study makes these structural and thermal changes seem to be highly complicated and inconclusive, where they can be caused directly by action of boric acid or the reactive boron-containing spices resulting from the interaction of methanol with the boric acid or indirectly by the interactions of boron-containing compounds with uncured or cured epoxy resin. H_3_BO_3_ eliminates water at a temperature higher than 100 °C. The water released during its decomposition reduces the temperature and can dilute the combustible gases in the combustion zone^38^. At higher temperatures, H_3_BO_3_ is converted to boric trioxide (B_2_O_3_) which can form a glassy film on the burning surface of the polymer, thus inhibiting the diffusion of flammable gases in the combustion zone and reducing the flame spread on the polymer surface^7^. On the other side, the potential intra-molecular B–N interactions that would form complexes such as 1,3,6,2-dioxazaborocanes (DOABs) (cross-linking), and formation a Lewis acid/base type products as well as hydrogen bonding, can also enhance the thermal stability of epoxy resins.

#### DSC thermograms

Differential scanning calorimetry (DSC) was used to determine phase transition temperatures and glass transition temperature (*T*_*g*_) of plain and B-doped ER specimens prepared using varying BA proportions under non-isothermal linear heating (10 °C/min) from ambient temperature to 800 °C.

**Figure 5:**
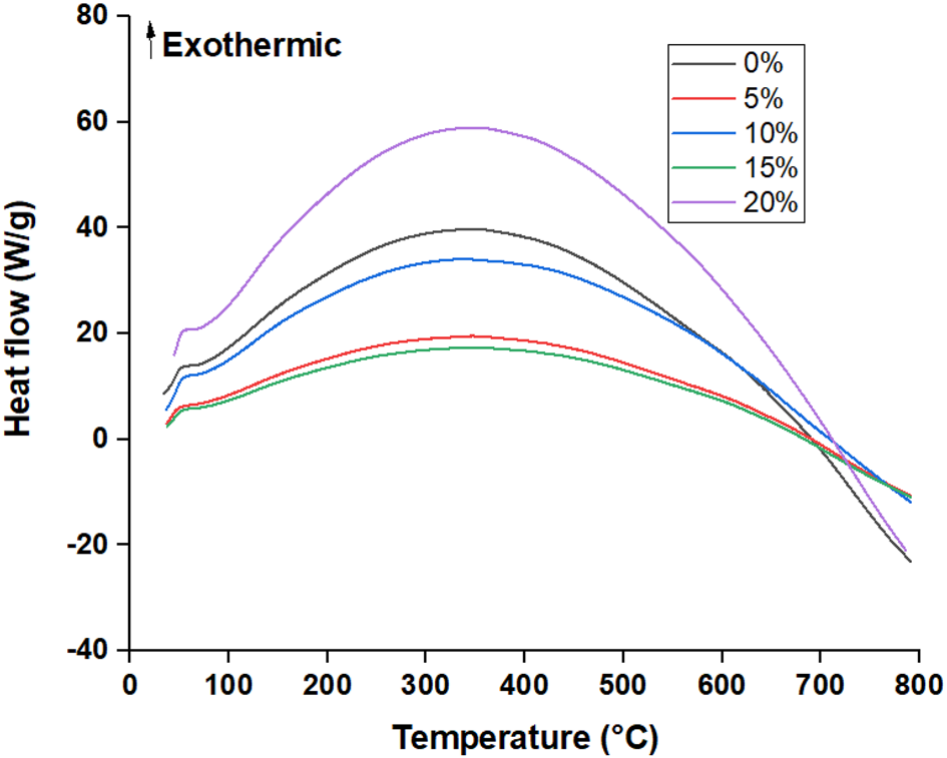
DSC thermograms of plain ER and B-doped ER specimens prepared at varying boric acid concentrations.

DSC thermograms of plain ER and B-doped ER specimens are shown in Fig. 5. These thermograms of both typical and boron-modified epoxy resin exhibited a curing exothermic peak confirming that the reaction doesn’t go to completion in the first heating run (during resin fabrication) hence on heating a second time, the post-curing can be observed. These exothermic peaks reveal that the reaction between the epoxy resin and the hardeners in the absence or presence of boron–containing compounds occur with interfering physical transitions followed by beginning of decomposition. Moreover, the curing exothermic peaks reflect the level of reactivity and epoxide cure conversion of DGEBA. Where, the start of the curing reaction is shifted to a lower temperature of boron-doped epoxy resin specimens. In comparing with plain epoxy resin, the decomposition exothermic peaks of heat flow became smaller with reduced height but stay almost around 350 °C with incorporating boric acids. This can be attributed to the potential reactions between boric–containing species and epoxy resin with no significant weight loss.

Glass transition temperature (*T*_g_) is widely used to indicate the curing degree that depends on its chain flexibility, cross-linked structure, and intermolecular hydrogen bonding of the resin ^39,40^. However, *T*_max_ indicates the speed of the curing process. *T*_*g*_ and *T*_*max*_ of plain and boron-modified epoxy resin are tabulated in Table 4. These findings showed the glass transition temperature (*T*_g_), the inflection point of the curing exothermic peak, was increased with increasing the proportions of BA up to 15%. This increase in the glass transition temperatures can be explained by that the boron-containing compounds react with epoxy resin rather than the hardener (a higher degree of cross-linking). In this context, it has been previously reported that the glass transition temperature of thermosetting plastics such as epoxy resin shifts to higher temperatures at higher degrees of cross-linking^41^. Contrary to that, incorporating a higher amount of boric acid in the epoxy formulation led to a slight decrease in the *T*_*g*_ of the boron-doped epoxy resin specimen compared to the neat epoxy resin specimen. Similarly, the thermal decomposition process of boron–modified epoxy resin specimens has one stage as the pristine epoxy resin. The *T*_*max*_ of plain epoxy resin was ~ 353 °C. t is found that the incorporation of BA up to 15% in epoxy resin increased *T*_*max*_ by ca. 11 °C, in comparison with that of pure epoxy. The higher thermal stability might be because the BA incorporating increases the cross-link density of the epoxy. However, the *T*_*max*_ was decreased by incorporating boric acid in the epoxy resin at a higher amount. This increase in the cross-linking density of these boron-modified epoxy resin specimens (5–15%) can be supported by increasing the hardness with increasing the amount of boric acid incorporated in the epoxy resin as mentioned above. This can be attributed to the homogenous distribution of boron-containing species because of the high miscibility of epoxy resins system components resulting in increasing the cross-linking density that can enhance the ductility of the polymer matrix. Notwithstanding, declining the cross-linking density at the highest BA concentration (20%) might be due to inhibition of the curing and subsequently lowering the cross-linking density.

**Table 4:** *T*_g_ and *T*_*max*_ of plain ER and B-doped ER specimens.

Boric acid (%)	0	5	10	15	20
*T*_*g*_ (°C)	90.50	92.83	94.40	97.49	88.26
*T*_*max*_ (°C)	353.58	357.85	361.12	364.25	326.29

#### DTA thermograms

**Figure 6:**
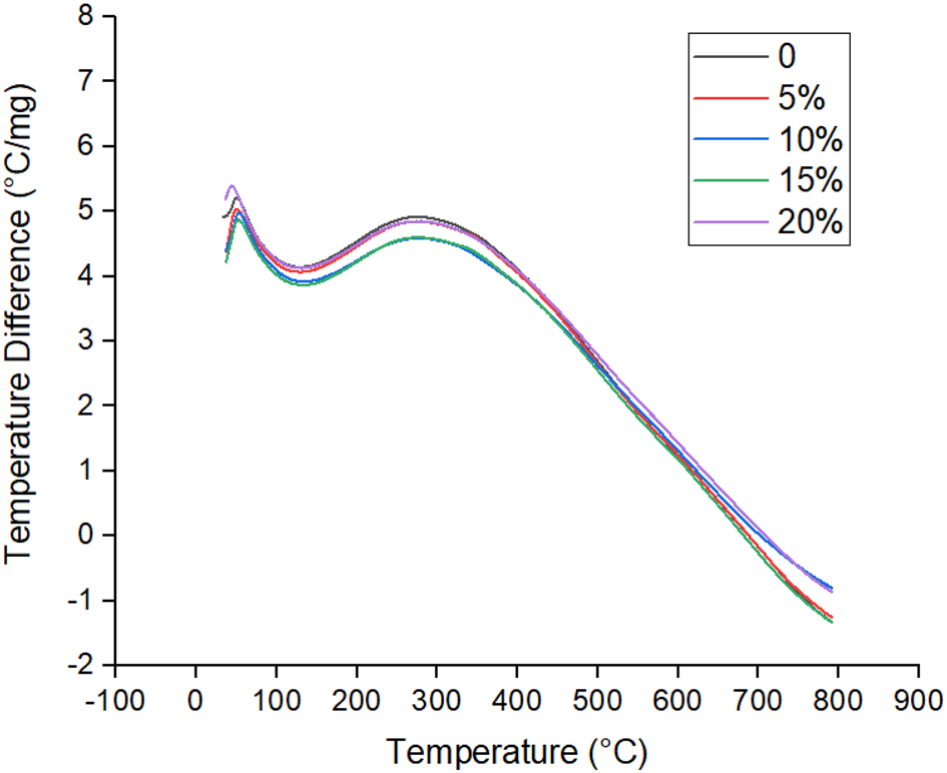
DTA thermograms for plain ER and BA-doped ER specimens prepared at varying boric acid concentrations.

Differential thermal analysis (DTA) was conducted for plain ER and B-doped ER specimens to explore the characteristic exothermic peaks and measure the melting point. The DTA thermograms were demonstrated in Fig. 6 and the data obtained from these thermograms were given in Table 5. DTA thermograms of plain ER and B-doped ER with varying boric acid concentrations exhibited two exothermic peaks. The first peak belongs to the residual curing and completion of rearrangements of network segments, and the second to epoxy resin thermo-oxidation^42^. The second exothermic peak has particular importance because it represents the thermal decomposition of the resin. Therefore, the higher onset temperature of second endothermic event (*T*_*on2*_) reflect the high thermal stability of the polymer^42^. From these findings, it was indicated that the onset temperature of the second exothermic peak (*T*_*on2*_) and the melting point of B-doped ER specimens were increased by increasing the concentration of the boric acid up to 15%. These results proved that incorporation of boric acid in the epoxy resin formulations enhance the thermal stability of the resin specimen.

**Table 5:** DTA representative temperatures for plain ER and B-doped ER specimens.

Boric acid (%)	*T*_*on1*_ (°C)	*T*_*on2*_ (°C)	Melting point (°C)
0	51.55	273.24	114.63
5	50.46	277.72	118.24
10	52.97	278.21	121.98
15	52.14	290.65	125.60
20	43.87	272.78	112.31

#### Autoclaving endurance

**Figure 7:**
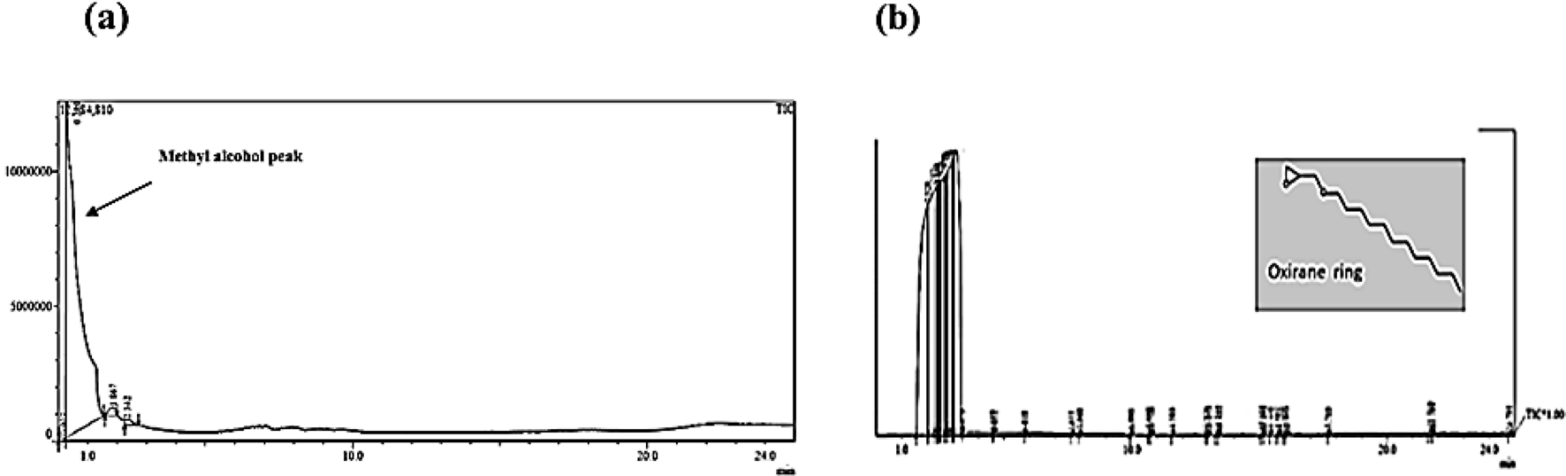
GC–MS chromatograms of migrants from boric acid-doped epoxy resin specimens in different simulants; distilled water (**a**) and dichloromethane (**b**).

Sterilization of the microfluidic devices utilized in most biological applications such as organs-on-chips employed in biomedical applications is a crucial process to avoid contamination which can affect the validity of experiments and the accuracy of the results. Hence, the ability of these devices to endure such a sterilization process is a key property in these applications^24,43^. Due to the advantages of steam sterilization (autoclaving) including the high efficiency, ease of use, low cost, and no use of toxic compounds^44^, B-doped ER-based microfluidic platform (prepared from a composition containing 10% BA) was sterilized in an autoclave at 121 °C for 20 min under the pressure of 2 atm. as performed on the plain epoxy resin-based microfluidic plate in our previous study for assessing the serviceability of these micro-plates based on the modified epoxy resin under autoclaving conditions with the comparison with plain ER-based ones. The gross weight the fabricated B-doped ER micro-plate and dimensions, and surface roughness of the micro-plate and their micro-channels were measured and the data of these trails are tabulated in Tables 6 and 7. Further, 3D micrographs for these micro-plates before and after autoclaving are presented in Fig. 7. These results showed that there are no significant differences in the parameters used for evaluation of B-doped ER-based micro-plates and their micro-channels. Besides, 3D micrographs of B-doped ER-based chips exhibited no formation of bulges as a result of the autoclaving process. Likewise, the same results have been exhibited in the case of ER-based microfluidic microchips fabricated in our previous study, but for their micro-channels, the autoclaving process significantly lowered the height of bulges previously formed around the rim of grooves during the micromachining. Accordingly, boron-doped epoxy resin-based microfluidic chips have good endurance for autoclaving process, and subsequently, they can potential candidate in biological assays.

**Table 6:** Impact of autoclaving process on the dimensions and surface roughness of the B-doped ER-based microfluidic micro-plates.

Parameter	Before autoclaving	After autoclaving
Weight (gm)	2.25 ± 0.25	2.25 ± 0.25
Length (cm)	3.5 ± 0.2	3.5 ± 0.2
Width (cm)	3.5 ± 0.1	3.5 ± 0.1
Thickness (mm)	23 ± 0.1	23 ± 0.1
*Ra* (µm)	1.25 ± 0.268	1.11 ± 0.058

**Table 7:** Impact of autoclaving on the dimensions and surface roughness of CO_2_ laser-engraved micro-channel on B-doped ER-based microfluidic micro-plates.

Parameter	Before autoclaving	After autoclaving
Width (µm)	96.68 ± 0.68	86.94 ± 3.05
Depth (µm)	95.95 ± 3.21	92.53 ± 2.24
*Ra* (µm)	9.83 ± 1.27	10.61 ± 0.29

#### Overall and specific migration

**Figure 8:**
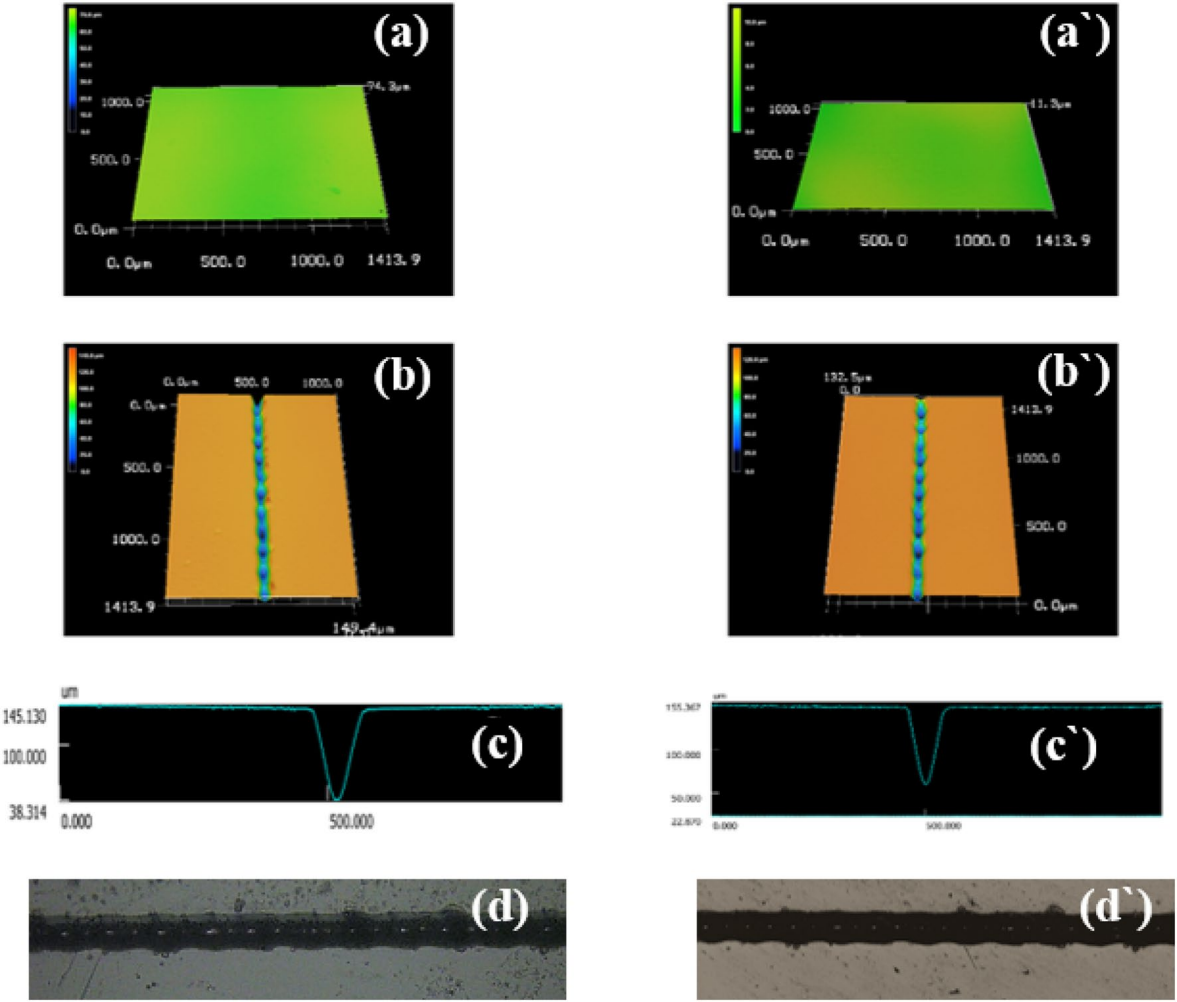
3D micrographs of BA-doped ER-based micro-plate (10% boric acid) before (**a**) and after autoclaving (**a***'*), 3D micrographs and profile of micro-channel engraved at a laser power of 2.4 W and speed of 5 mm/s at 10% before autoclaving (**b** and **c**, respectively) and after autoclaving (**b*'*** and **c*'***, respectively), and optical microscope micrograph of the same micro-channel before (**d**) and after autoclaving (**d*'***).

Polymer substrates of the microfluidic platforms have to don't release any components that would interfere with or inhibit the sensitive compounds employed in the intended analysis, particularly those in the biomedical and biotechnological fields such as polymerase chain reaction (PCR)^45^. Therefore, determination of the overall and specific migration is so essential. Regarding the polar solutions of PCR and the high denaturation temperature of PCR (95 °C), migration testing has been conducted using distilled water as a stimulant at 100 °C^46^. The overall migration of the B-doped ER specimen in polar simulant (water) was less than 0.1 mg dm^−2^. The GC–MS chromatogram is shown in Fig. 8a. This chromatogram has just revealed one peak that refers to the solvent (methanol), indicating no presence of other substances transferred from the B-doped ER specimen into the polar simulant, distilled water. Accordingly, these boron-doped epoxy resin-based chips can be employed in DNA amplification and other biomedical applications. The overall migration for B-doped ER specimens measured in dichloromethane (DCM) simulant was 5.3 mg dm^−2^. This less polar simulant exhibited higher potency for occurring migration than the more polar counterpart (distilled water). In the GC–MS chromatogram (Fig. 8b), the migration was dominated by a derivative containing one oxirane ring (1 EPO), a hydrolysis product of bisphenol A diglycidyl ether.

#### PCR compatibility

PCR is a thermal cycling procedure to amplify target DNA. PCR components one at least would come into contact with the surface of the reaction chamber because a PCR mixture is composite. The binding of PCR components to the material surface will disrupt the delicate and optimized concentrations of the PCR components, which will have a detrimental effect on the PCR performance. Surficial interactions may obstruct the biochemical components, slowing or, in the worst case, completely inhibiting the reaction^47^. PCR inhibition can also occur by inhibitory components that may leach from the substrate of the microfluidic device into the PCR mixture^48^. The compatibility of the materials employed in microfluidic fabrication with PCR has to be addressed as an urgent issue. The PCR compatibility with B-doped ER-based chips was assessed and the results were listed in Table 8. These findings exhibited a noticeable incompatibility for both of plain ER and B-doped ER-based chips with PCR. In this context, it is worth mentioning that the overall and specific migration testing showed that no migrants were detected in the simulant. Therefore, the mechanism of PCR inhibition via the inhibitory impact of components released from the epoxy resin can be excluded and thus, the interactions of PCR components with epoxy resin substrate could play a major role in incompatibility issues. To overcome this challenge, there are two main methodologies have been developed including a passive coating of the inner surface of the reaction chamber or the micro-channel, or a dynamic coating that involves adding components to the PCR mix that will minimize contact between the PCR components and the surrounding surfaces^48^. Herein, the incorporation of BSA or coating of B-doped ER-based chips with BSA was tried. The compatibility assessment results of B-doped ER-based chips with PCR after these treatments (Table 8) showed that application of BSA as a liquid coating on surface of plain ER-based chips and B-doped ER-based chips exhibit higher compatibility with PCR. This higher compatibility can be attributed to the act of BSA in covering these polymer plates and consequently minimizing the adsorption of PCR mix ingredients, especially the DNA polymerase, onto the plate surface. On the other hand, incorporating BSA into the PCR mixture had a lower impact in enhancing the compatibility of these plates with PCR compared with the BSA coating. This lower impact can be due to BSA being thought to compete with DNA polymerase on active adsorption sites on the chip surface, thus blocking polymerase adsorption to some extent^48^.

**Table 8:** PCR Compatibility of plain ER-based chips and B-doped ER-based chips after coating with BSA or incorporating BSA in PCR mixture.

Specimens and treatments	Boric acid (%)				
	0	5	10	15	20
B-doped ER-based chips	–	–	–	–	–
BSA-coated B-doped ER-based chips	++	++	++	++	++
B-doped ER-based chips with incorporating BSA in PCR mix	+	+	+	+	+

### Micro-channel dimensions

#### Micro-channel width

**Figure 9:**
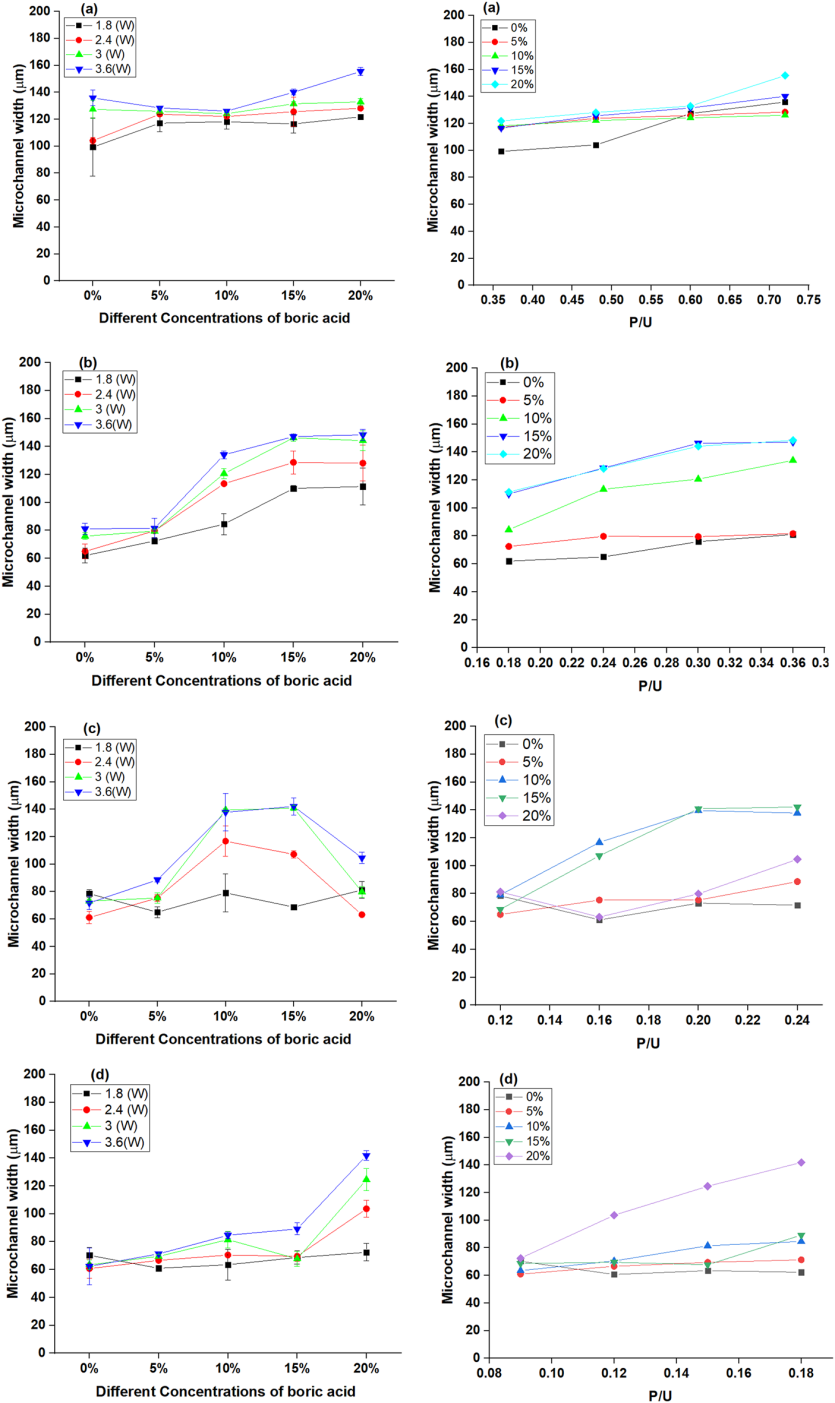
Effect of varying concentrations of boric acid; 5, 10, 15 and 20% (at the left) and P/U (at the right) on micro-channel width at different scanning speeds; 5 mm/s (**a**), 10 mm/s (**b**), 15 mm/s (**c**), and 20 mm/s (**d**).

The effect of laser input parameters and the proportions of boric acid on the micro-channel width are shown in Fig. 9. As illustrated from these results, the width of micro-channel CO_2_ laser ablated on plain ER- and B-doped ER chips increased with increasing laser power at all tested scanning speeds. It was decreased while the scanning speed was increased to 20 mm/s. These findings are in agreement with those previously reported by Prakash and Kumar on thermoplastic polymethylmethacrylate (PMMA)-based microchips^22^. Further, increasing the boric acid concentration increased the micro-channel width. The micro-channel was depicted with the highest width by high-output lasers moving slowly i.e., at 3.6 W and 5 mm/s. Whereas the highest micro-channel width on the plain epoxy resin (~ 135.91 μm) was recorded at a scanning speed of 5 mm/s and laser power of 3.6 W. So, the highest micro-channel width on B-doped ER chips (20% boric acid) (~ 155.53 μm) obtained at the same processing parameters. Hence, compared to direct parameters, the composed parameter, laser output power over moving speed (P/U), may have a greater impact on micro-channel width (Fig. 9).

#### Micro-channel depth

**Figure 10:**
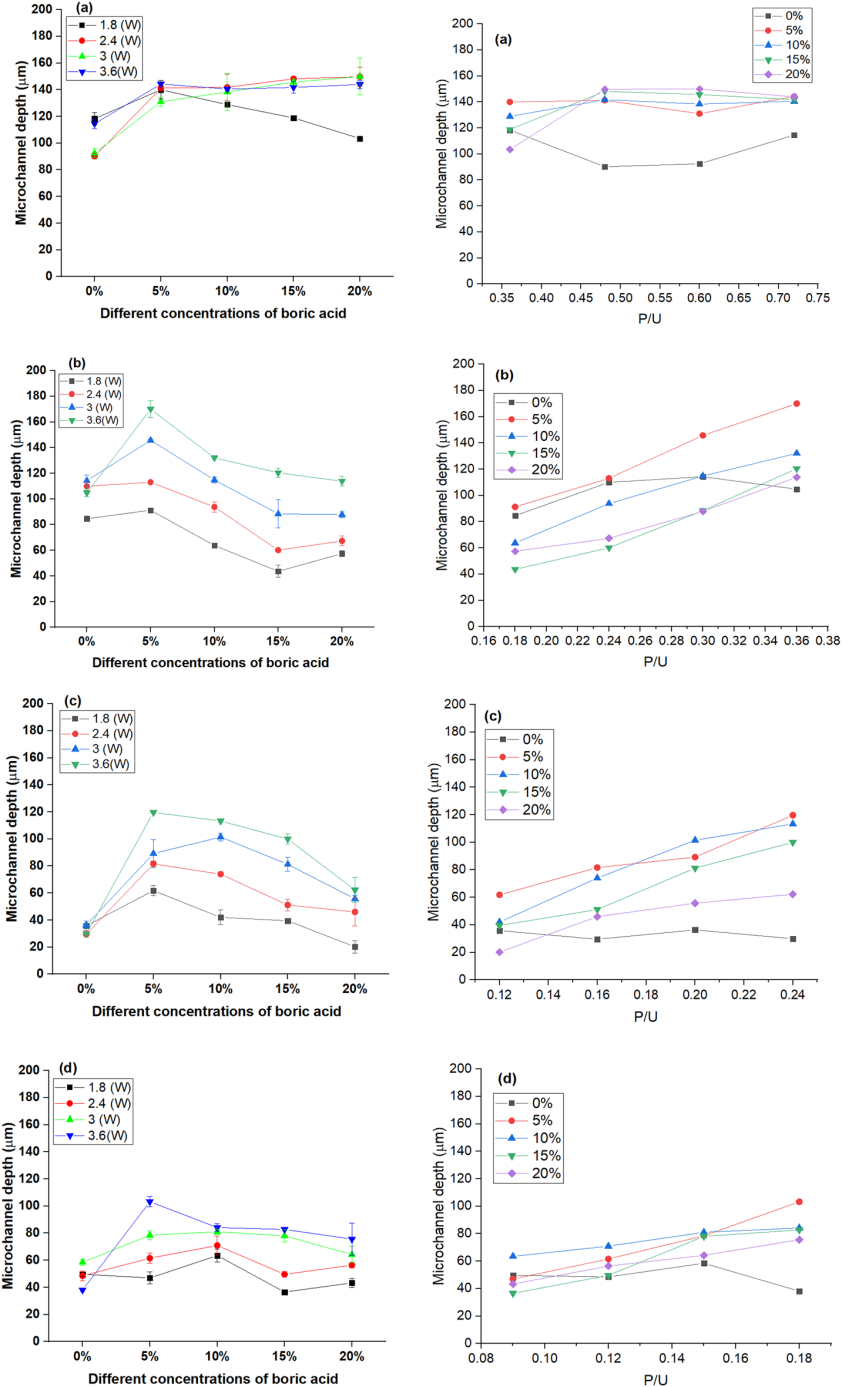
Effect of varying concentrations of boric acid; 5, 10, 15 and 20% (at the left) and P/U (at the right) on micro-channel depth at different scanning speeds; 5 mm/s (**a**), 10 mm/s (**b**), 15 mm/s (**c**), and 20 mm/s (**d**).

The impact of laser input parameters and boric acid concentration on the micro-channel depth is demonstrated in Fig. 10. It was realized that increasing the laser power increased the micro-channel depth at all tested scanning speeds. Moreover, the micro-channel depth was increased with increasing the proportion of boric acid. However, the micro-channel depth of B-doped ER plates was higher than that of plain ER-based plate. This increase in the micro-channel depth for B-doped ER can be attributed to increasing the crosslinking density of the epoxy resin matrix by an act of boric acid which increases its stiffness and subsequently the resistance of the epoxy substrate for the laser beam. Notwithstanding, the depth of the micro-channel was decreased by increasing the scanning speed from 5 to 20 mm/s. At the lesser speed, the substrate will be exposed to the laser beam for a longer time, and consequently, it absorbs a higher amount of the laser energy, acquiring more heat and consequently leading to increasing the depth of the groove^49,50^. The highest micro-channel depth (~ 170.03 μm) was obtained at a laser power of 3.6 W and a scanning speed of 10 mm/s for microchips prepared by 5% boric acid in the epoxy composition. Meanwhile, at 1.8 W and 15 mm/s, the lowest micro-channel depth of about 20.25 m was recorded for microchips prepared by incorporating 20% of boric acid in the epoxy composition. The relationship between P/U and micro-channel depth was plotted and displayed in Fig. 10. It was observed that the depth increased as P/U was increased.

#### Aspect ratio

**Figure 11:**
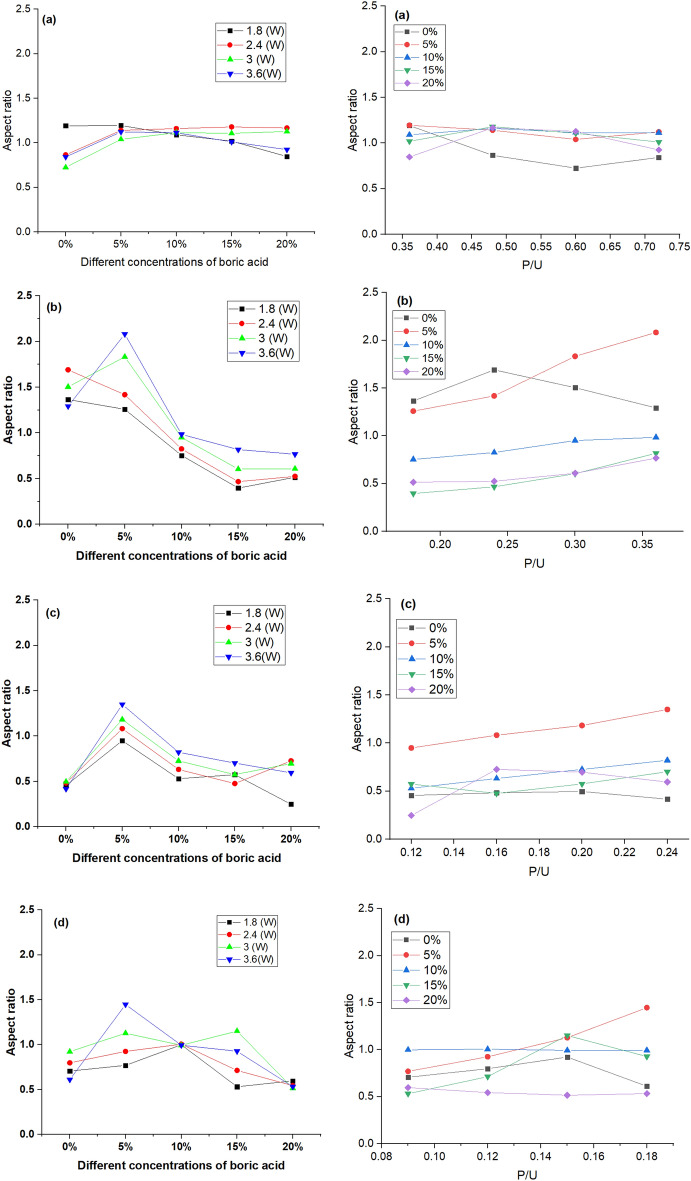
Effect of varying concentrations of boric acid; 5, 10, 15 and 20% (at the left) and P/U (at the right) on aspect ratio at different scanning speeds; 5 mm/s (**a**), 10 mm/s (**b**), 15 mm/s (**c**), and 20 mm/s (**d**).

The product of dividing micro-channel depth by micro-channel width is defined as the aspect ratio. A high aspect ratio of a micro-channel is considered a key feature in the technical feasibility of microfluidic chips, particularly in life science, to combine cell screening action with high fluidic throughput in continuous flow systems^51^. Figure 11 depicts the effect of input power and scanning speed on the aspect ratio. The findings showed that the micro-channels engraved onto B-doped ER chips prepared using 5% BA have the highest aspect ratios at all tested speeds and powers, and the highest value (~ 2.08) was recorded at 10 mm/s and 3.6 W. However, the aspect ratio of the micro-channel drawn at the same parameters over plain ER-based chips was ~ 1.292. At a boric acid concentration higher than 5%, the aspect ratio was reduced by increasing the proportion of boric acid. This decline in aspect ratio can be explained by enhancing crosslinking density for the epoxy resin matrix resulting from accelerating the curing process and the interactions of epoxy resin and boric acid during the fabrication. This can be confirmed by increasing the specimen’s hardness. Therefore, penetration resin surface with the laser beam became difficult. Also, it was indicated that by raising the P/U parameter, the aspect ratio increased.

#### Micro-channel quality

##### Micro-channel roughness

**Figure 12:**
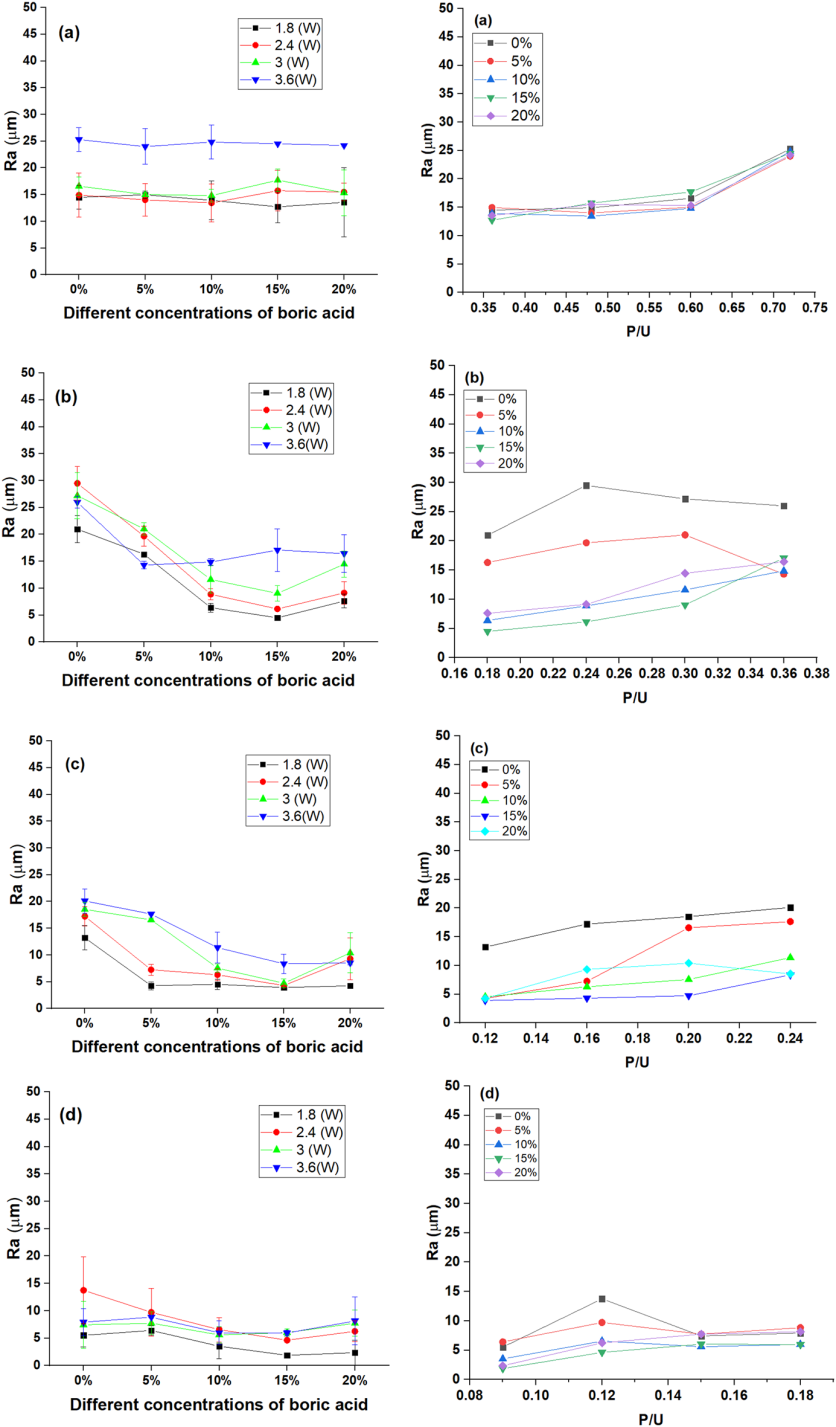
Effect of varying concentrations of boric acid; 5, 10, 15 and 20% (at the left) and P/U (at the right) on surface roughness of micro-channel at different scanning speeds; 5 mm/s (**a**), 10 mm/s (**b**), 15 mm/s (**c**), and 20 mm/s (**d**).

The roughness of the micro-channels surface is a crucial issue for its high impact on fluid flow and the efficiency of the microfluidic devices. The arithmetic average of roughness (*R*_*a*_) was chosen as a measure of surface roughness because it is the average arithmetic height of surface irregularities (peaks and valleys) from the mean line along the scanning length at the bottom of the micro-channel^52^. The influence of input power, scanning speed, and BA concentration incorporated into ER matrix on the *R*_*a*_ of the micro-channel surface is shown in Fig. 12. It was indicated that the surface roughness, *R*_*a*_, increased with raising the laser power. However, it decreased by increasing the scanning speed. The highest roughness ~ 29.5 μm was recorded at a laser power of 2.4 W and a high scanning speed of 10 mm/s for ER-based chips. However, the lowest *R*_*a*_ (~ 1.88 μm) was recorded at laser power of 1.8 W and scanning speed of 20 mm/s for B-doped ER-based chips prepared using 15% boric acid.

At a scanning speed of 5 mm/s, no noticeable difference was recorded in the resulting surface roughness of micro-channel drawn over B-doped ER-based chips prepared with varying proportions of boric acid. The impact of boric acid concentration tends to be more pronounced at higher scanning speeds (> 5 mm/s) since the micro-channel surface roughness was reduced by increasing the boric acid concentration. These findings agree with those reported in a previous study that indicated that the roughness of the cut increased with decreasing laser scanning speeds^53^. Considering the impact of the composed parameter (P/U) that ranged from 0.35 to 0.75 on the roughness, it was indicated that by increasing P/U from 0.35 to 0.6, a slight increase in Ra. However, at higher P/U, a pronounced increase was observed. The 3D micrographs for plain ER- and B-doped ER-based specimens were given in S1 and S2.

##### Bulge height

The bulges on the rim of a micro-channel can be formed by two mechanisms. One is called the 'conventional bulge' caused by molten polymer re-solidification in a cool-air atmosphere. The other is referred to as a "hump" because of thermal distortion caused by thermal stress or residual stress in a large temperature gradient that forms on the rim of the micro-channel. Coating the microfluidic platform with PDMS or an unexposed JSR layer can eliminate the first type^54^. However, the last type cannot be removed and can induce cracks in the following processes.

**Figure 13:**
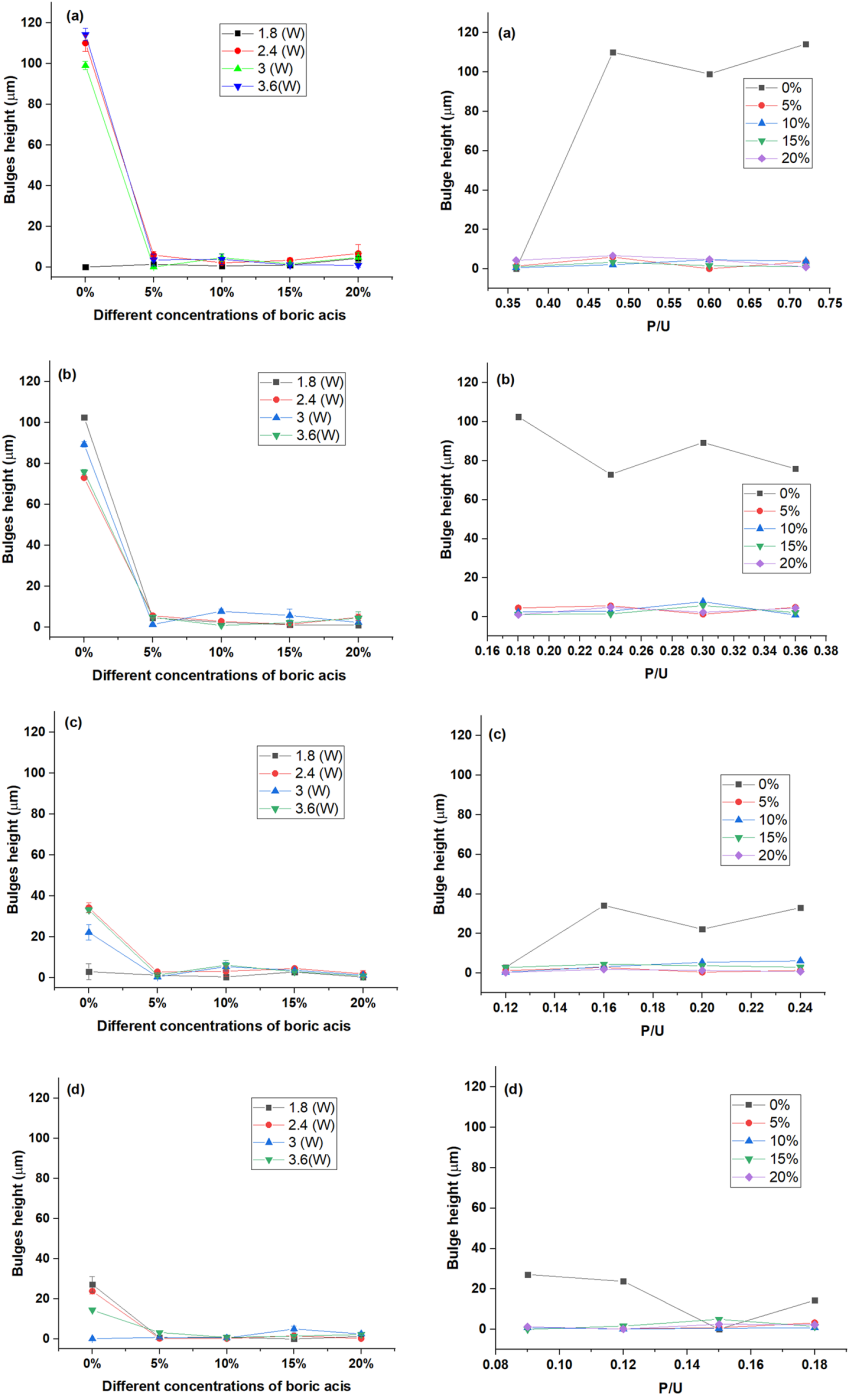
Effect of varying concentrations of boric acid; 5, 10, 15 and 20% (at the left) and P/U (at the right) on the bulge height formed onto micro-channel rim at different scanning speeds; 5 mm/s (**a**), 10 mm/s (**b**), 15 mm/s (**c**), and 20 mm/s (**d**).

The influence of laser ablation parameters and the amount of boric acid on the bulge height was investigated. The results are shown in Fig. 13. It was illustrated that increasing the laser power and slowing its speed led to increasing the bulge height for plain ER-based chips. The highest bulge height (~ 114.29 µm) was recorded for the neat ER-based platform (control) at laser power (3.6 W) and a scanning speed of 5 mm/s However, the lowest bulge height value of 0.027 was recorded at micro-machining parameters, 1.8 W and 20 mm/s. Herein, incorporating boric acid in the epoxy matrix at a concentration of 5% led to a more drastic effect on the bulge height than that for laser power and speed, as a noticeable reduction in the bulge height. However, a further increase in the proportions of boric acid by more than 5% has a slight impact on bulge height. The lowest bulge height value (0.022 µm) was recorded for B-doped ER-based chips prepared using 15% BA. Generally, the bulge height values obtained for all B-doped ER-based platforms fabricated with different proportions of BA were ≤ 6 µm. Regarding the negligible effect of the individual parameters on the bulge heights formed over B-doped ER-based platforms, the compound parameter, P/U, showed the same. These findings can be explained on the basis that the incorporation of boric acid can enhance the thermal stability of epoxy resin, i.e., by increasing the melting temperature, the conventional bulge formation was reduced to the minimum, and the ablated material would withstand higher thermal stress, which eliminates the thermal distortion and cracks formation^55^.

## Conclusion

Microfluidic devices based on novel boron-doped epoxy resin were successfully fabricated using casting and solvent evaporation followed by micromachining with mask-less, simple, and rapid CO2 laser ablation technique. Modification of epoxy resin compositions was achieved by incorporating H_3_BO_3_ after its dissolution in methanol into the epoxy resin matrix at different concentrations. Incorporation of BA was performed with the proposed to enhance the thermal stability of microfluidic substrate, and consequently, the dimension stability and quality of microfluidic micro-channels. The structural, physical, and thermal properties of the modified epoxy resin specimens were studied using FTIR and UV/Vis spectral analyses and mechanical testing, TGA, DSC, and DTA, and compared to those of the control unmodified (neat) epoxy resin specimen. Based on the finding of these studies, it was concluded that the mechanical and thermal stability of epoxy resin were noticeably improved via the acting of boron compounds formed and present in an epoxy matrix as a sintering and fluxing agent and simultaneous formation of DOPO derivatives and other potential interactions between ER and boron compounds. These improvements were fulfilled with a negligible effect on the optical transmittance of the epoxy resin chips. CO2 laser micro-machining has been operated using laser power in a range of 1.8–3.6W and scanning speed in the range of 5–20 mm/s to optimize the processing parameter of boron-doped epoxy resin and exploring the impact of the incorporation of boric acid in epoxy polymer compositions on the dimension and quality of engraved micro-channels. The findings of these trails showed a reduction in the surface roughness of micro-channels drawn over B-doped-ER micro-plates; the lowest roughness (~ 1.88 μm) was recorded for micro-plates fabricated at a laser power of 1.8 W and a high scanning speed of 20 mm/s with incorporating 15% BA. Also, a slight bulge height (≤ 6 µm) was obtained for all fabricated B-doped-ER micro-plates and the lowest value (0.022 µm) for BA-doped-ER fabricated at 1.8 W and 20 mm/s using 15% BA. In respect of the suitability of the fabricated B-doped ER-based microfluidic platform for bio-biomedical and analytical applications such as DNA amplification, it was demonstrated that these platforms have good autoclaving standability and PCR compatibility as well as no release for any components that would interfere with or inhibit the biological components employed in these analyses. This study validated the incorporation of boric acid into the ER matrix to enhance the thermal stability of the cured epoxy resin, which led to improved micro-the quality of the micro-channel with no negative impacts on the biological reactions. Therefore, the B-doped ER microfluidic devices developed in this study can be considered promising candidates for bio-analytical applications.

### Supplementary Information

Supplementary Tables.

## Data Availability

Correspondence and requests for materials should be addressed to Emad A. Soliman based on reasonable request.
