# Supplementary material for: Fabrication and characterization of microfluidic devices based on boron-modified epoxy resin using CO2 laser ablation for bio-analytical applications
Source: Sci Rep. 2023 Aug 3;13:12623. doi: 10.1038/s41598-023-39054-0 (PMC10400657; doi:10.1038/s41598-023-39054-0)
Supplement: Supplementary file 1 — Supplementary Tables. [file 41598_2023_39054_MOESM1_ESM.docx]

Table S1. 3D Laser microscope micrographs of laser ablated micro-channels over BA-doped ER

| 5 mm/s | 0% | 5% | 10% | 15% | 20% |
| --- | --- | --- | --- | --- | --- |
| 1.8W | 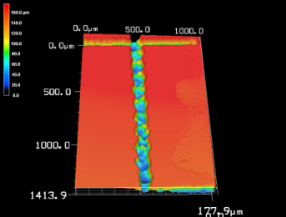 | 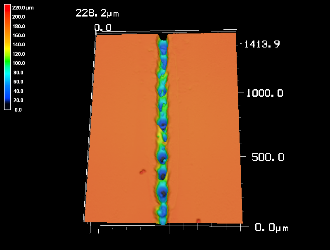 | 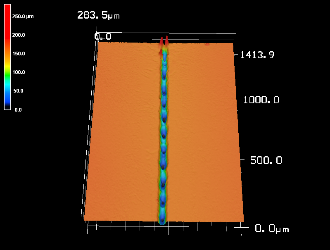 | 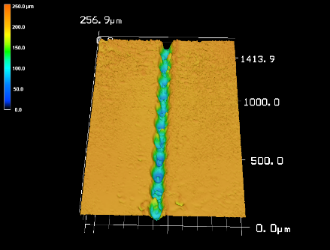 | 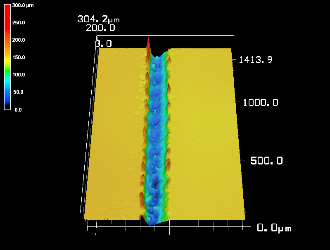 |
| 2.4W | 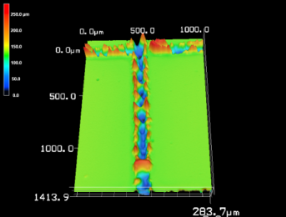 | 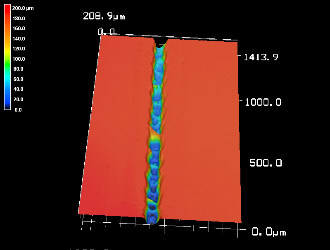 | 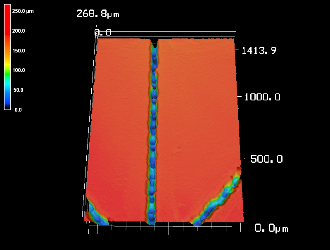 | 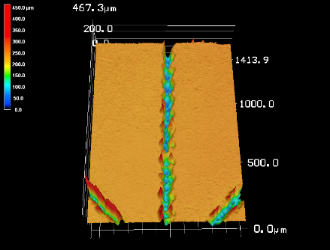 | 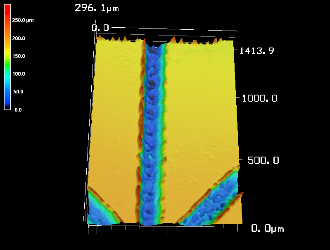 |
| 3W | 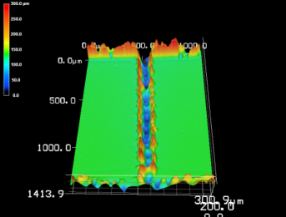 | 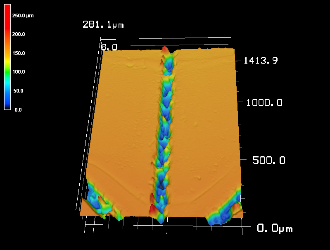 | 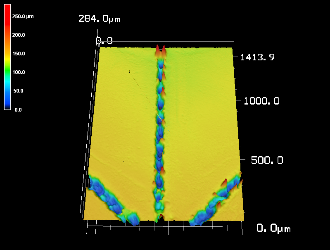 | 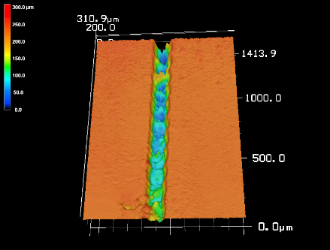 | 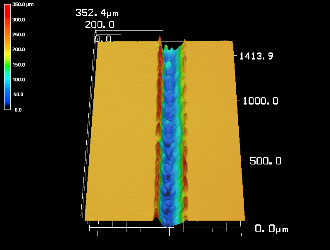 |
| 3.6W | 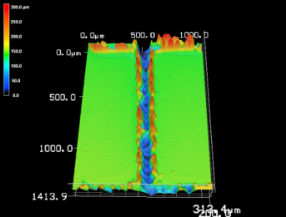 | 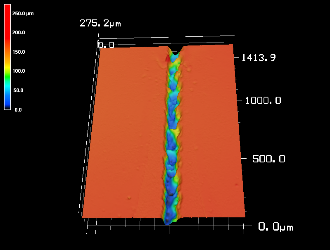 | 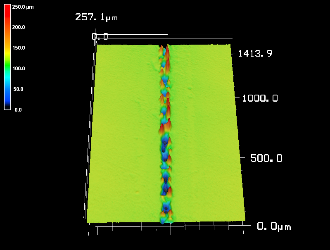 | 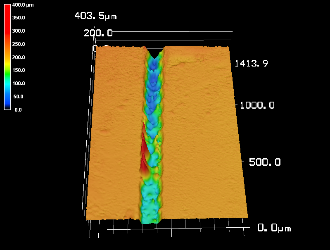 | 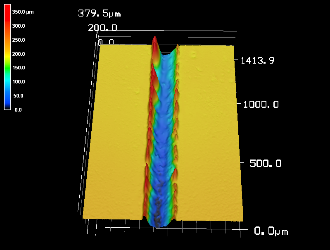 |
| 10 mm/s | 0% | 5% | 10% | 15% | 20% |
| 1.8W | 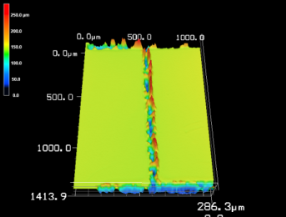 | 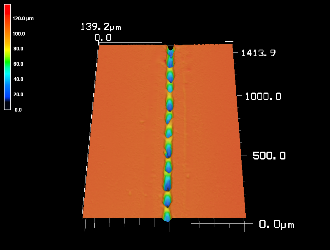 | 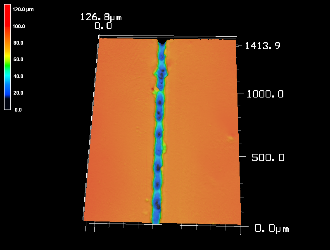 | 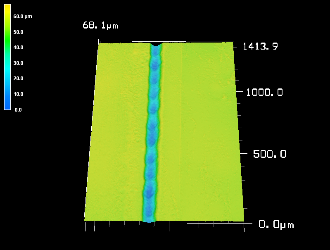 | 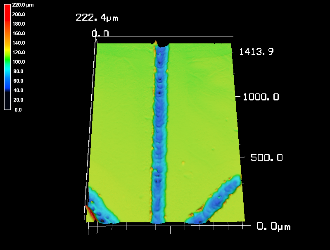 |
| 2.4W | 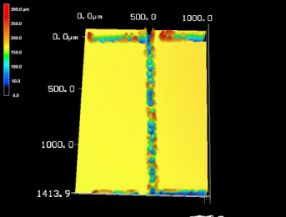 | 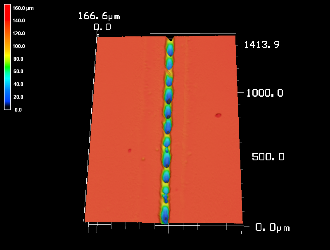 | 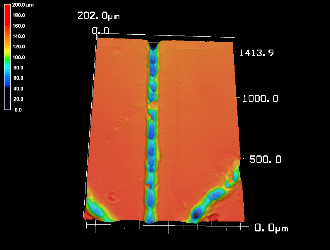 | 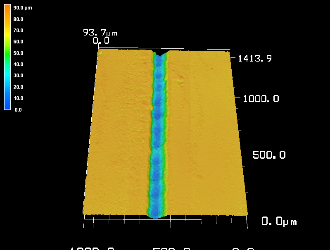 | 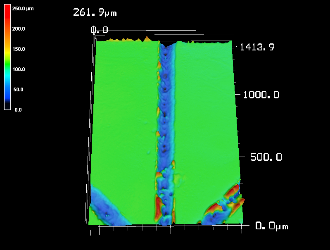 |
| 3W | 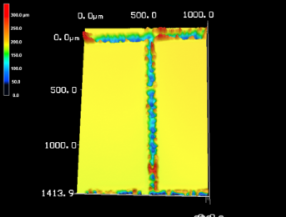 | 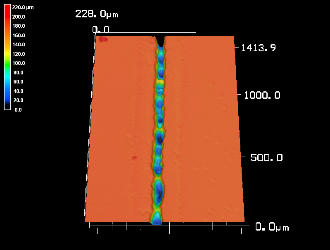 | 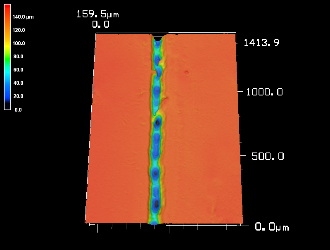 | 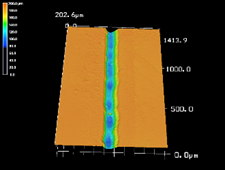 | 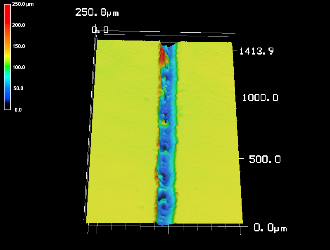 |
| 3.6W | 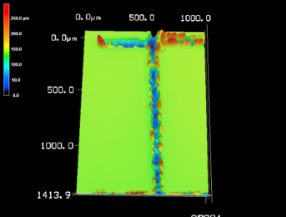 | 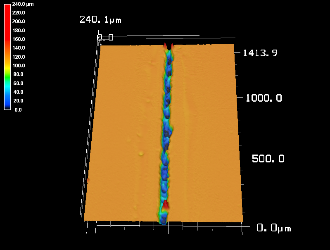 | 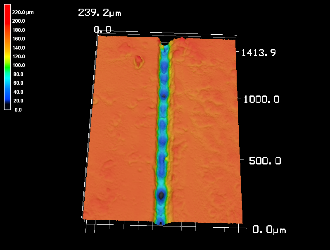 | 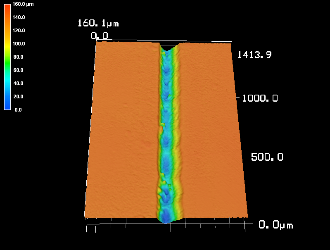 | 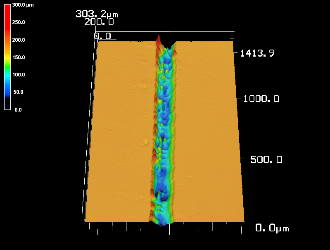 |
| 15 mm/s | 0% | 5% | 10% | 15% | 20% |
| 1.8W | 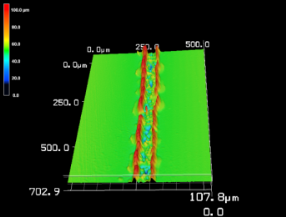 | 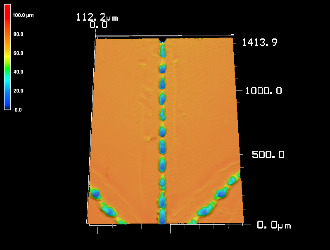 | 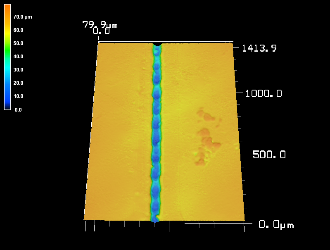 | **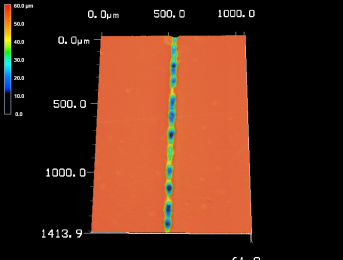** | 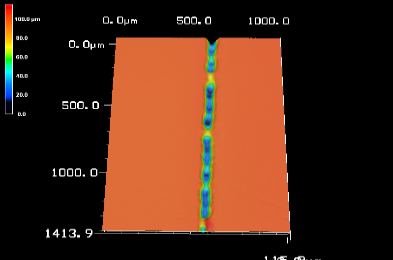 |
| 2.4W | 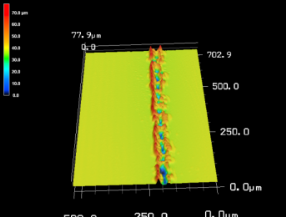 | 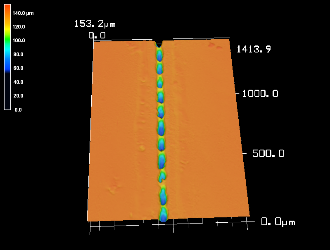 | 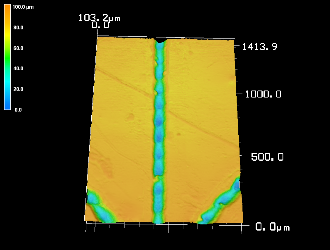 | 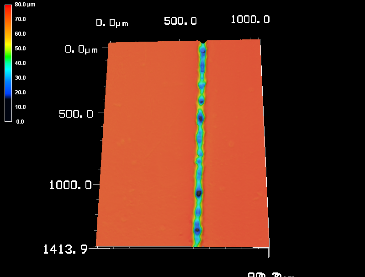 | 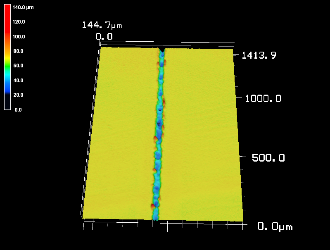 |
| 3W | 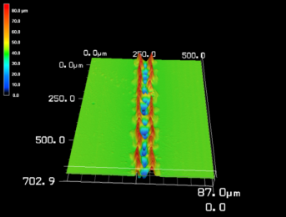 | 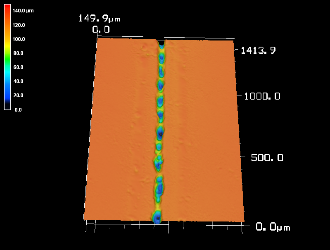 | 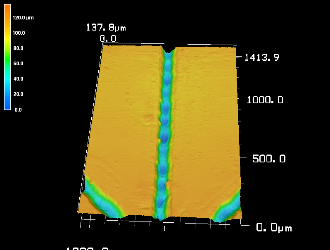 | 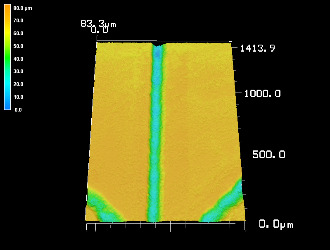 | 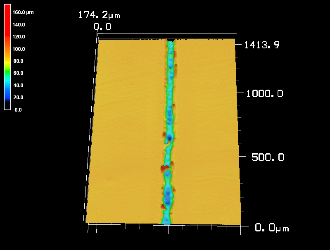 |
| 3.6W | 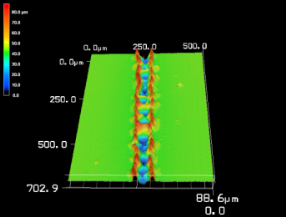 | 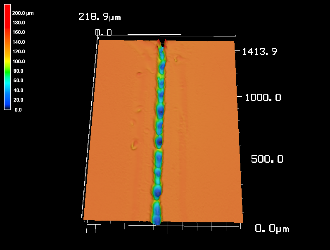 | 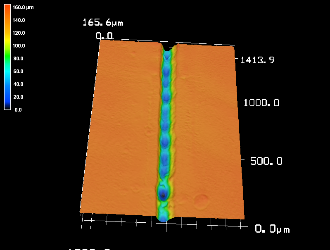 | 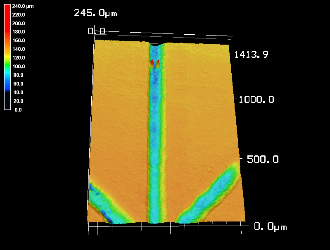 | 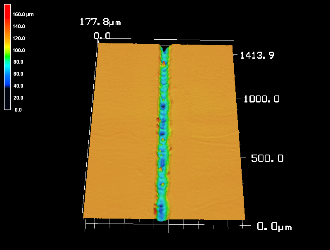 |
| 20 mm/s | 0% | 5% | 10% | 15% | 20% |
| 1.8W | 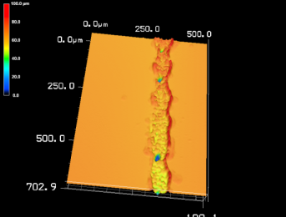 | 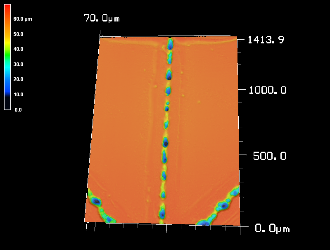 | 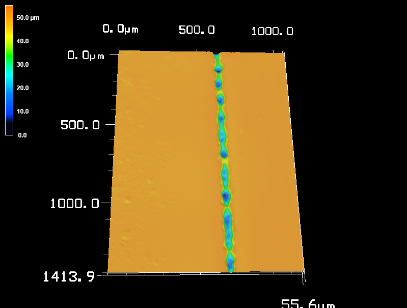 | 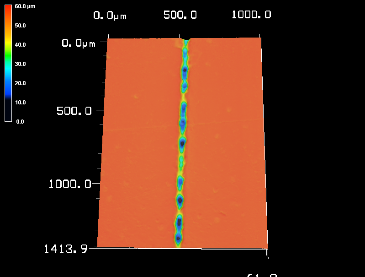 | 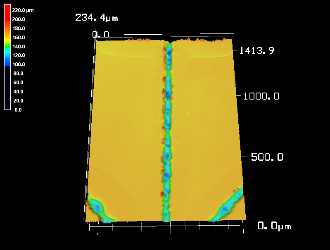 |
| 2.4W | 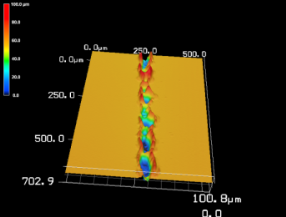 | 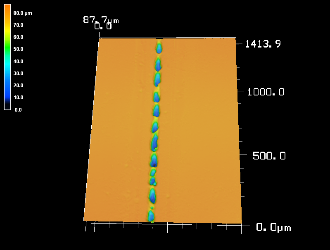 | 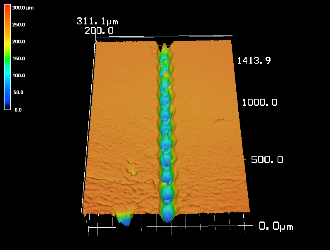 | 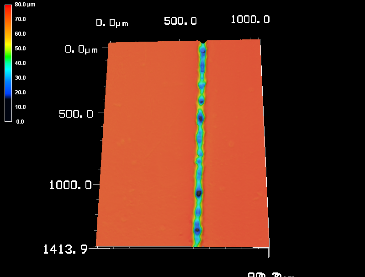 | 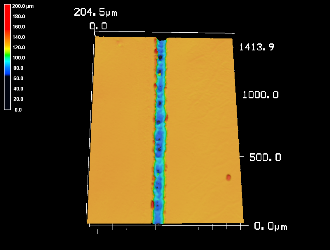 |
| 3W | 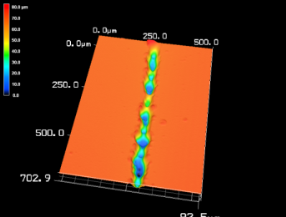 | 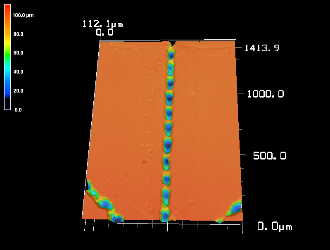 | 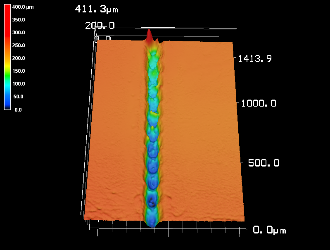 | 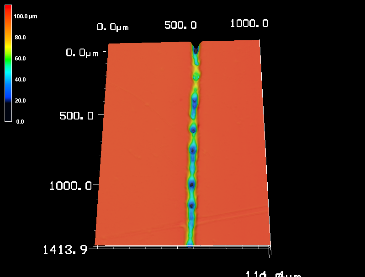 | 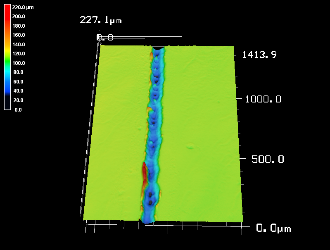 |
| 3.6W | 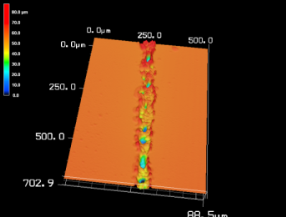 | 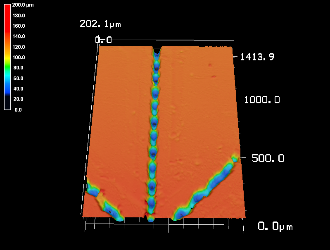 | 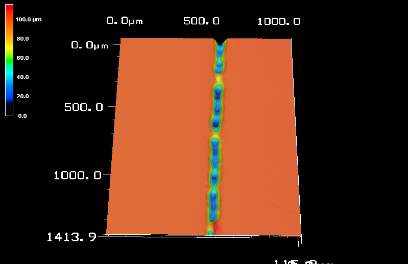 | 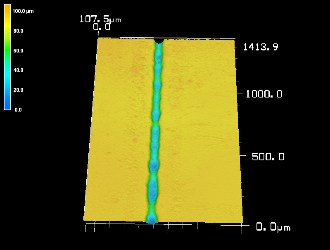 | 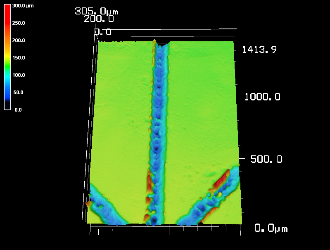 |

Table S2. Profile of laser ablated-micro-channel over BA-doped ER

| 5 mm/s | 0% | 5% | 10% | 15% | 20% |
| --- | --- | --- | --- | --- | --- |
| 1.8W | 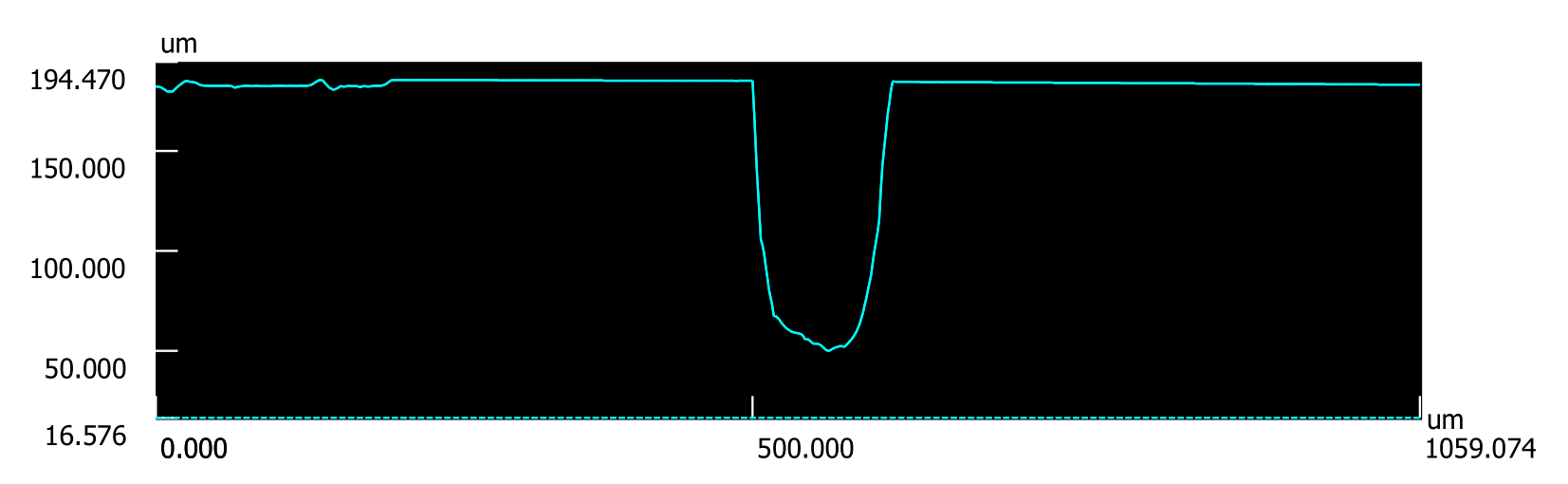 | 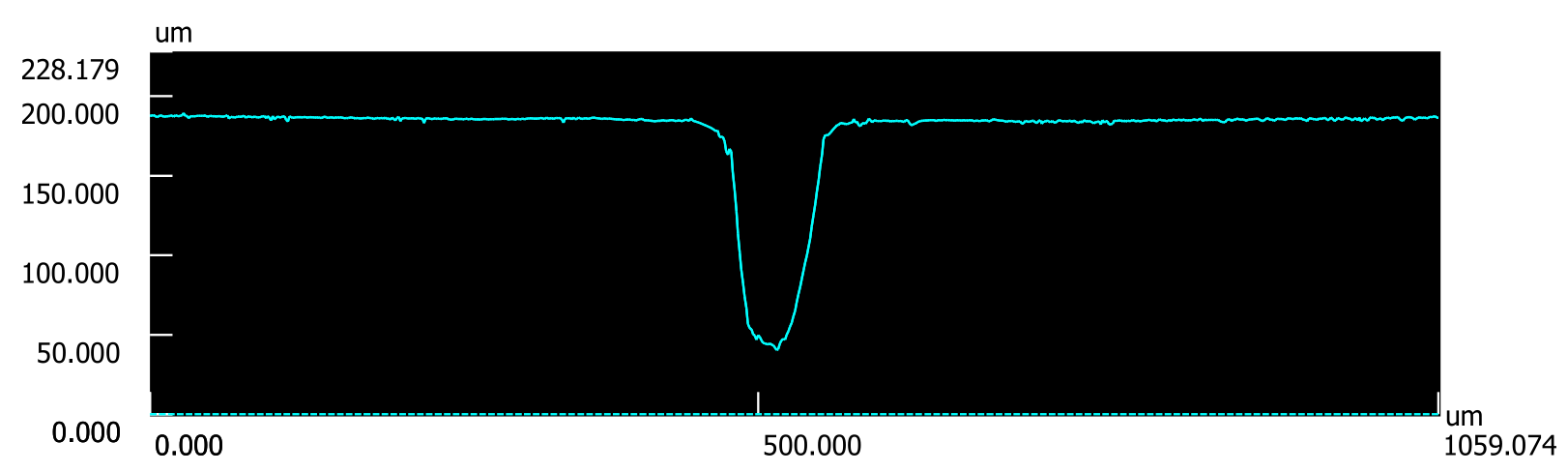 | 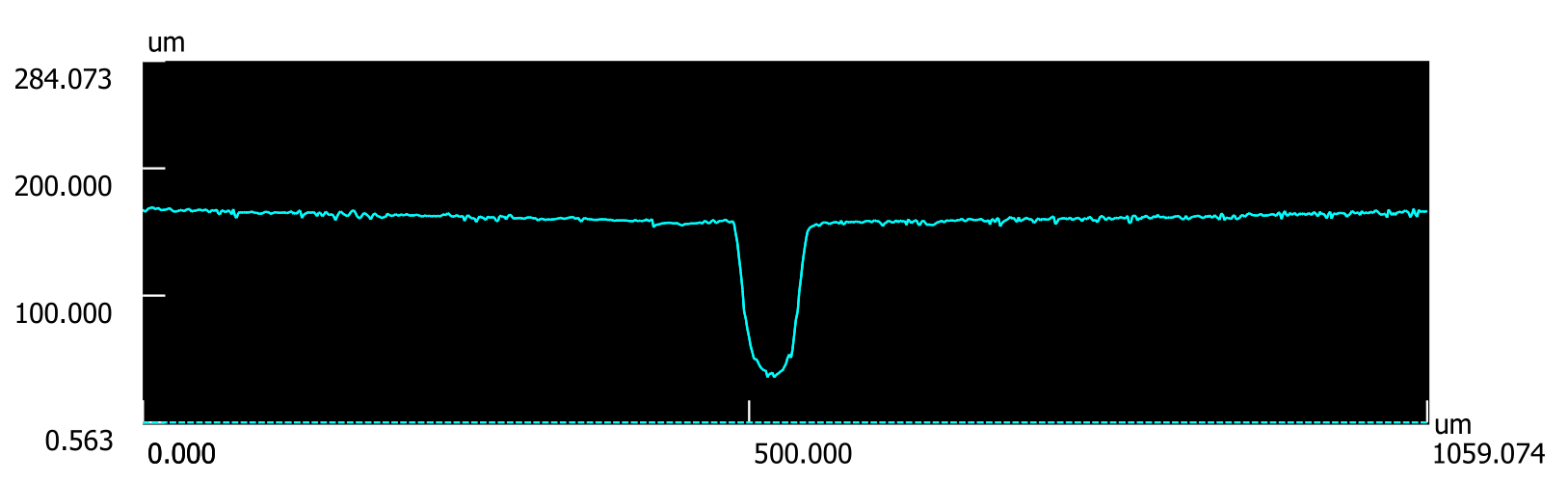 | 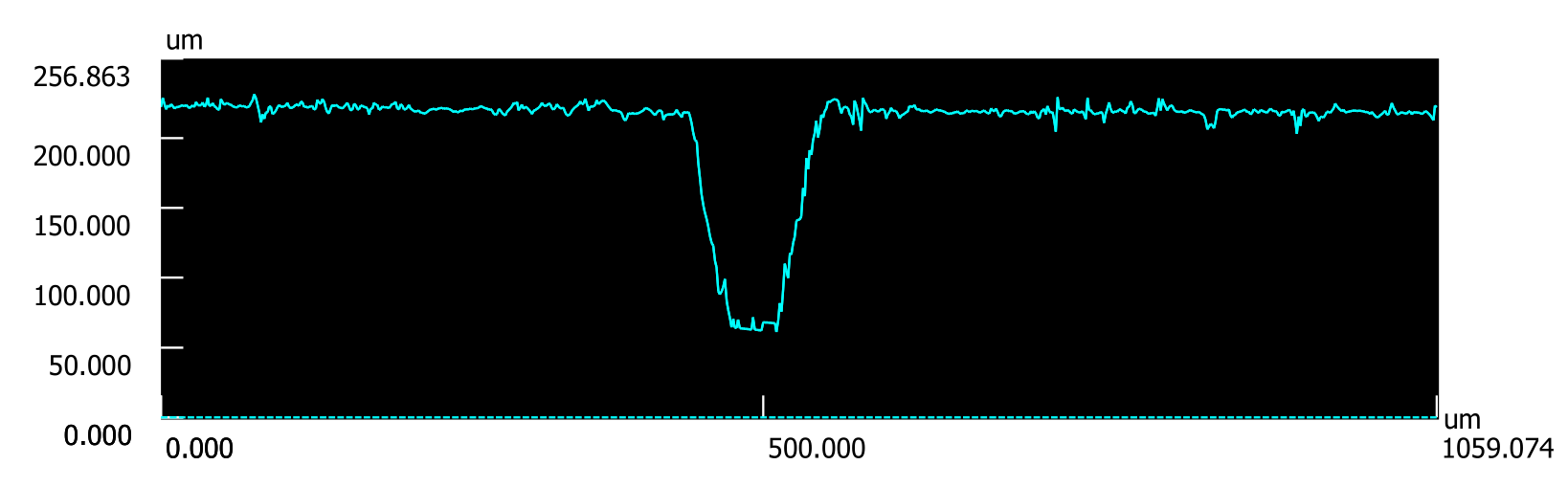 | 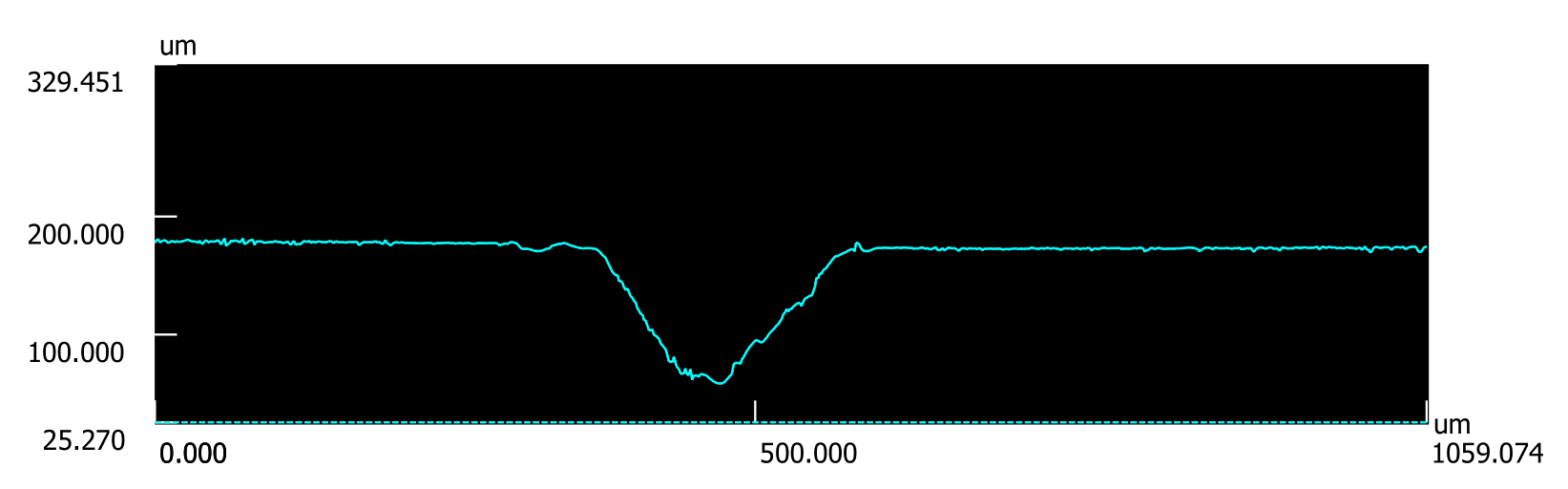 |
| 2.4W | 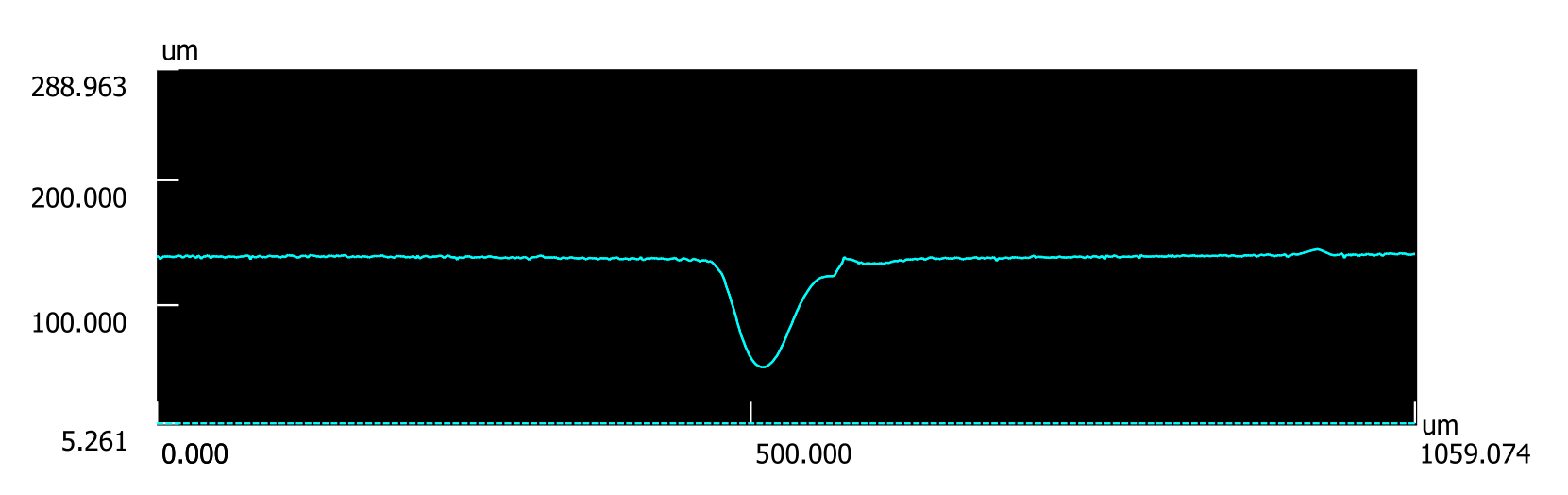 | 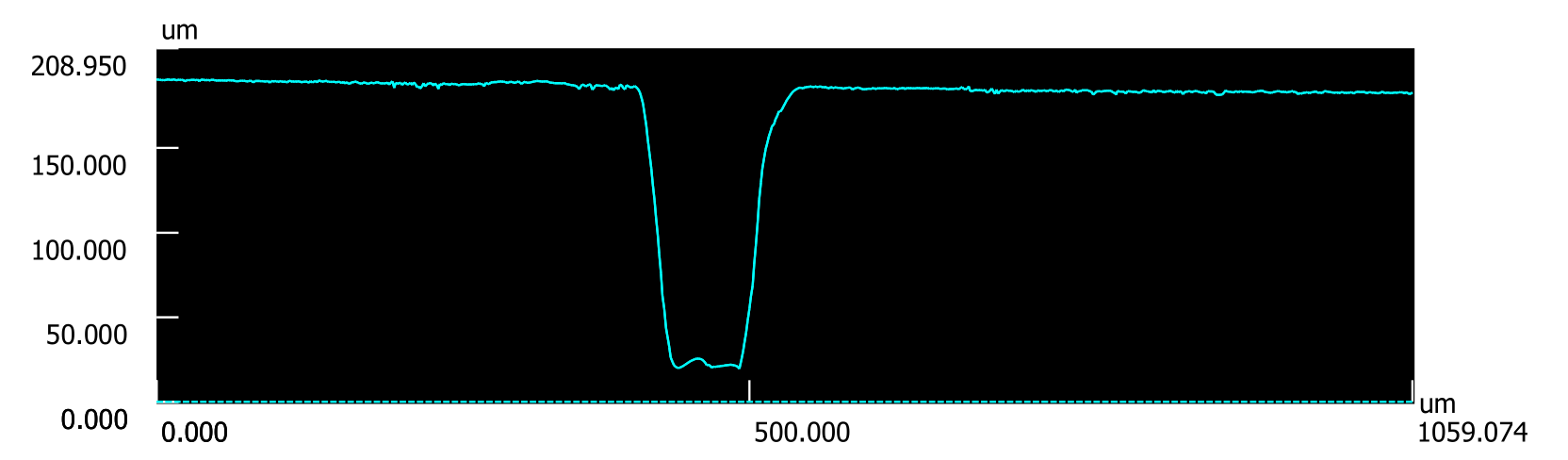 | 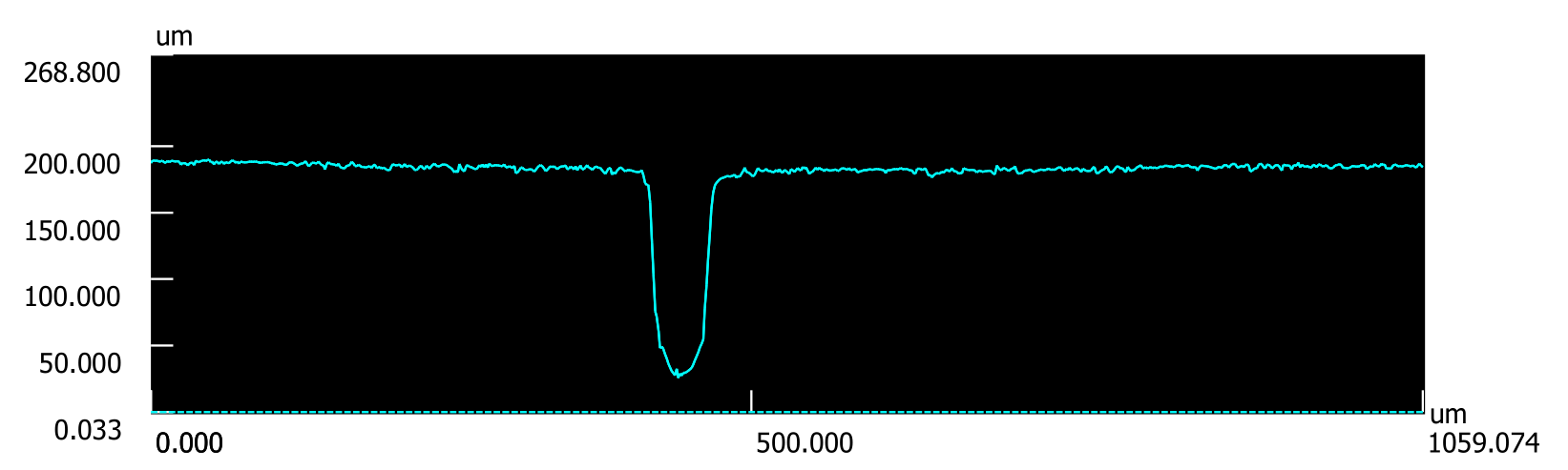 | 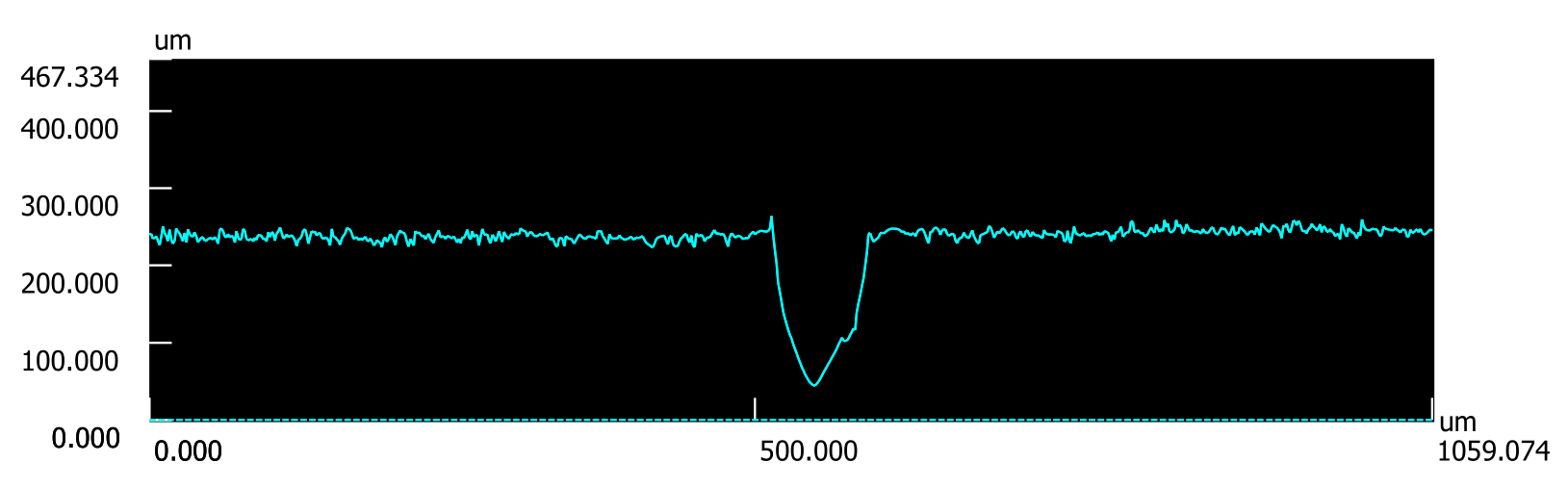 | 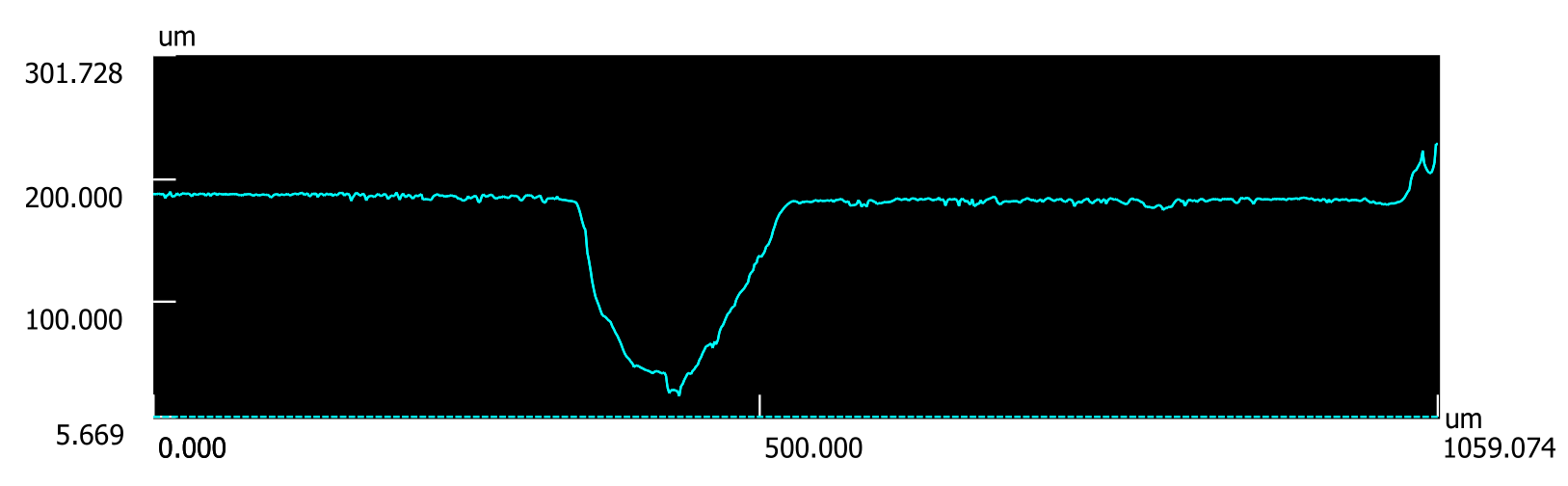 |
| 3W | 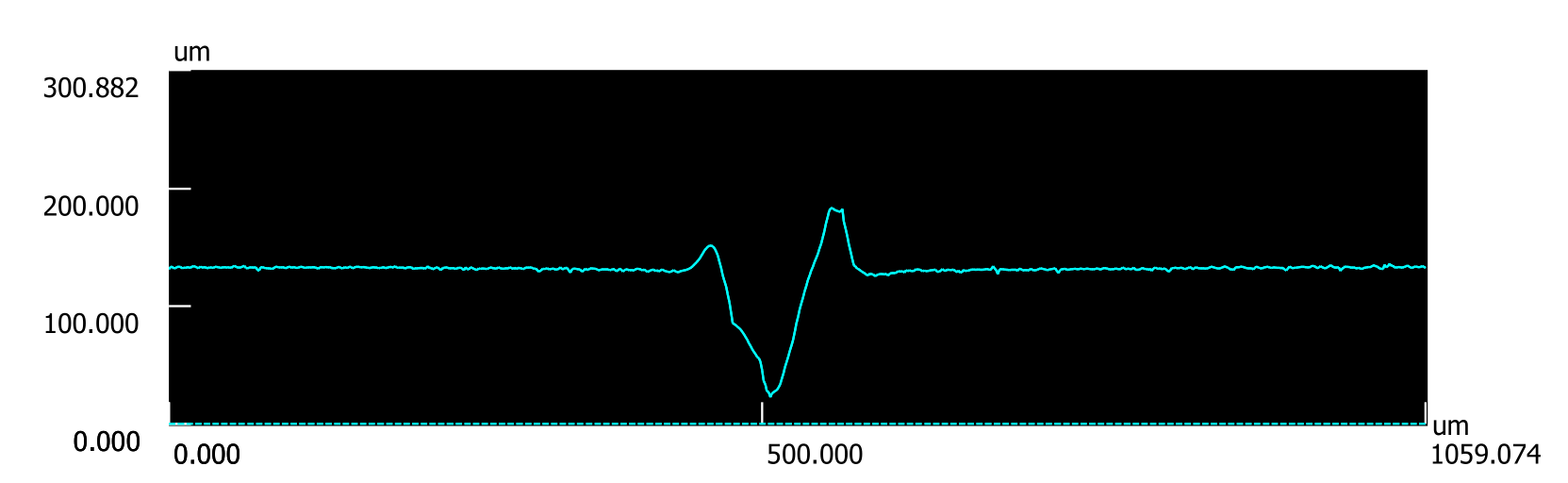 | 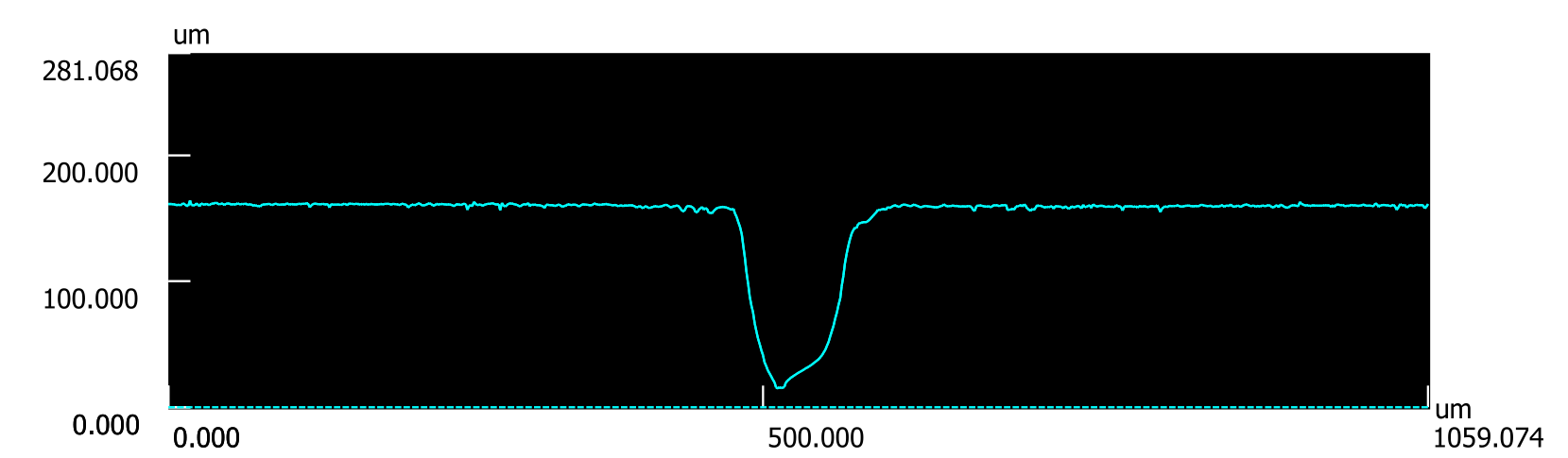 | 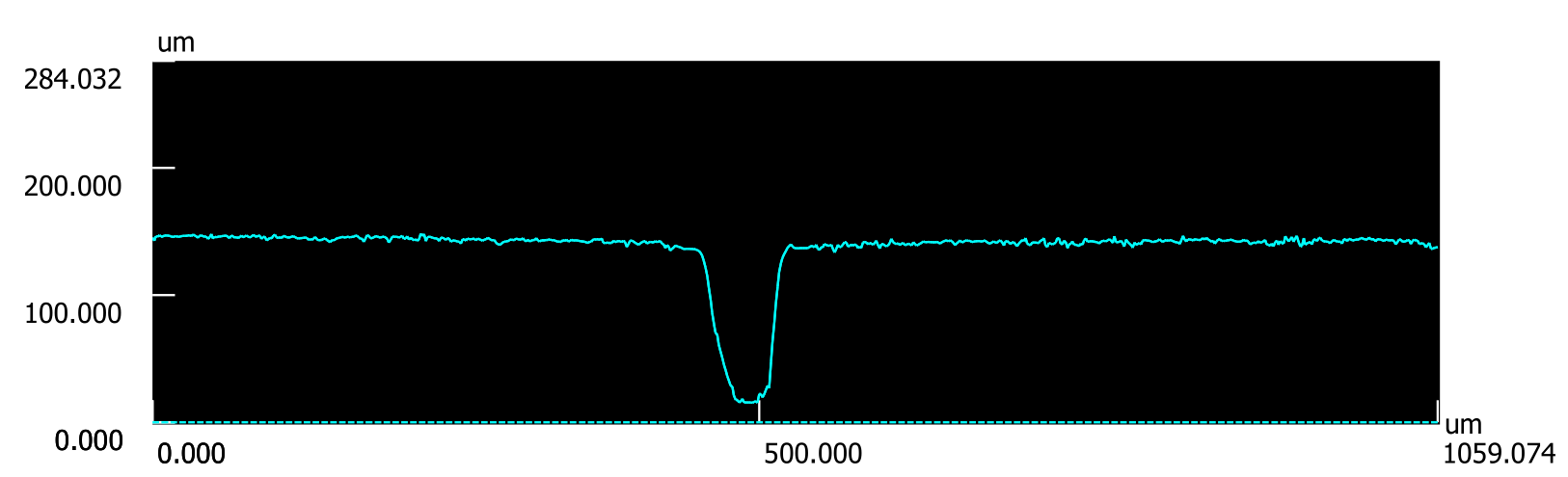 | 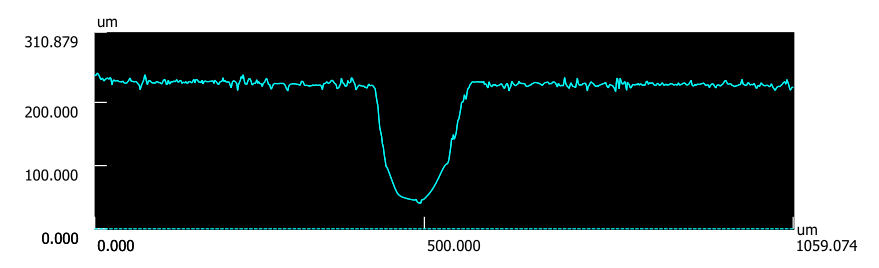 | 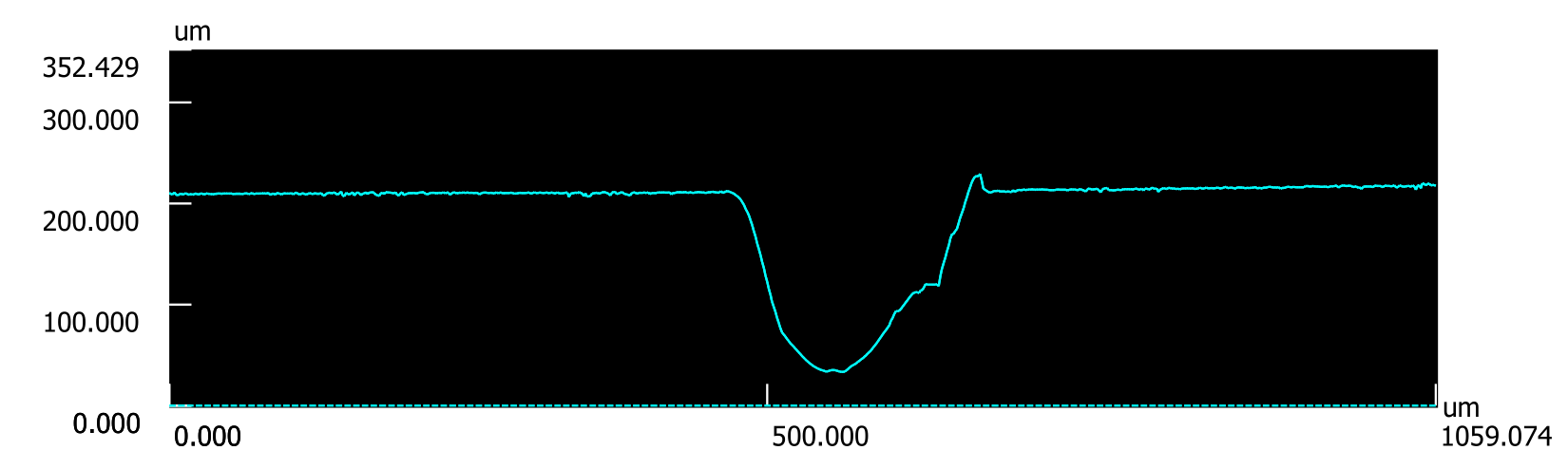 |
| 3.6W | 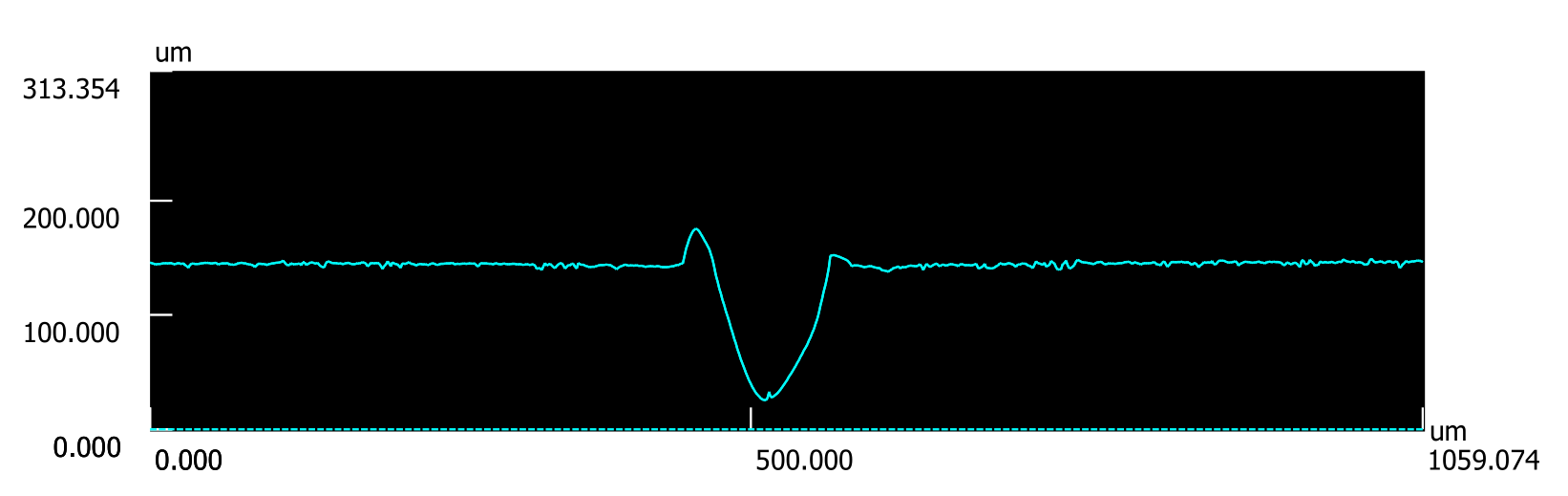 | 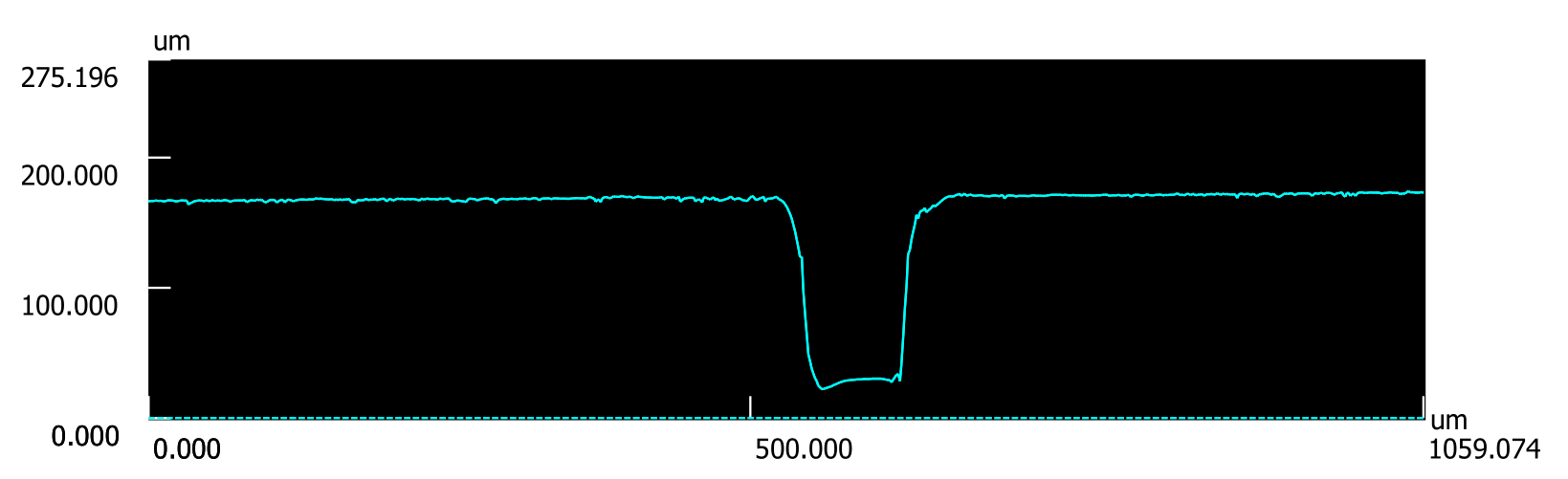 | 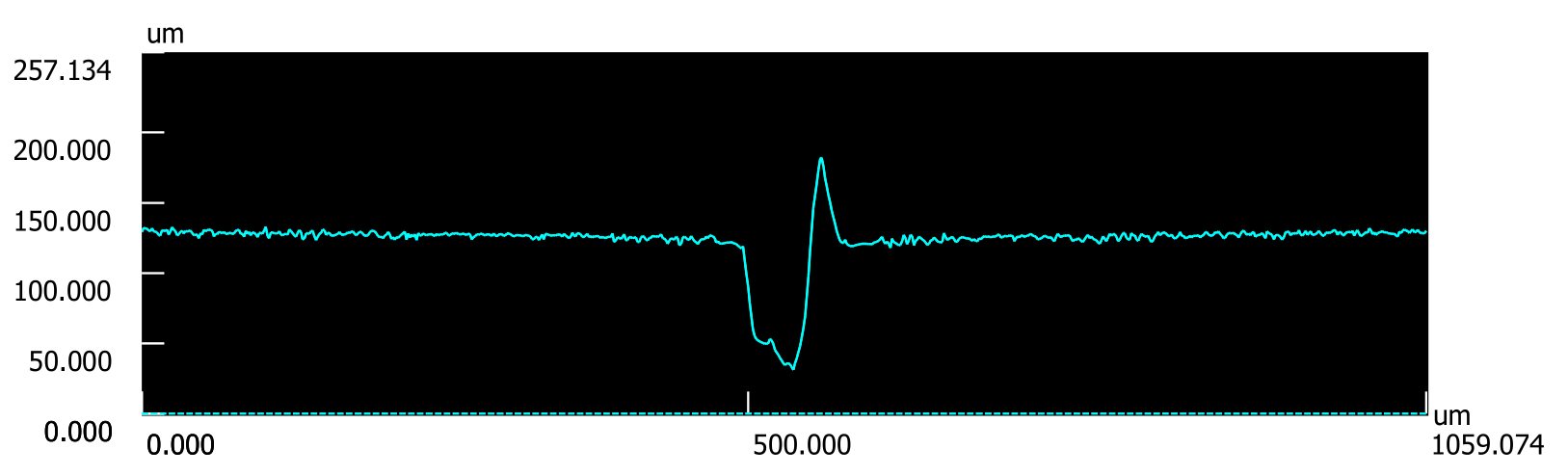 | 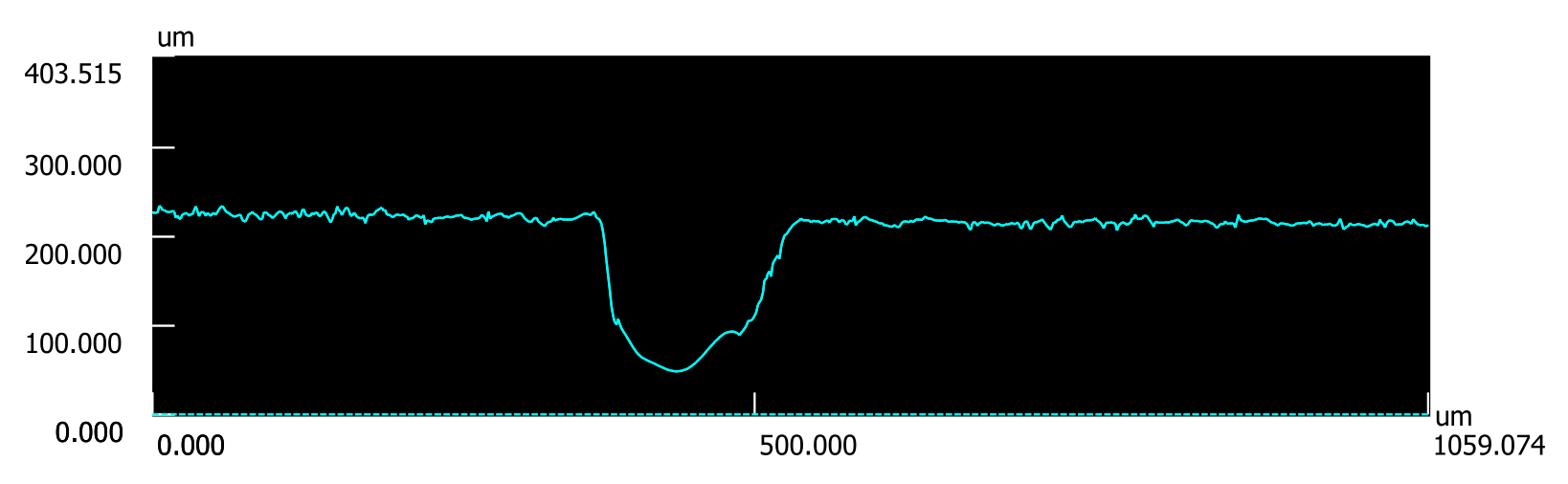 | 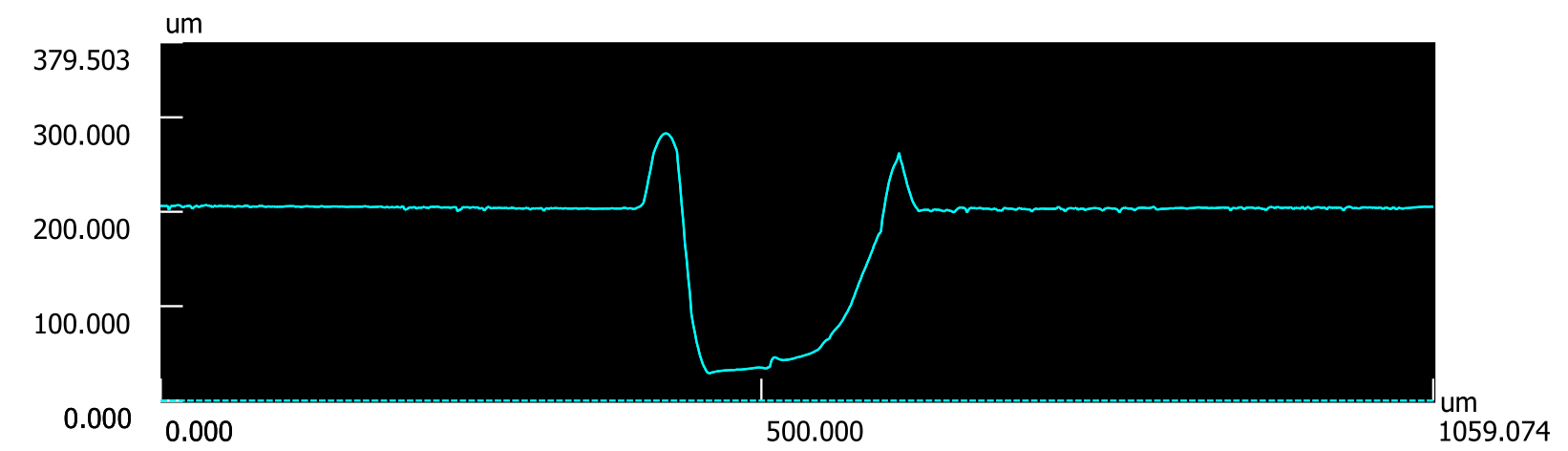 |
| 10 mm/s | 0% | 5% | 10% | 15% | 20% |
| 1.8W |  |  |  |  |  |
| 2.4W |  |  |  |  |  |
| 3W |  |  |  |  |  |
| 3.6W |  |  |  |  |  |
| 15 mm/s | 0% | 5% | 10% | 15% | 20% |
| 1.8W |  |  |  |  |  |
| 2.4W |  |  |  |  |  |
| 3W |  |  |  |  |  |
| 3.6W |  |  |  |  |  |
| 20 mm/s | 0% | 5% | 10% | 15% | 20% |
| 1.8W |  |  |  |  |  |
| 2.4W |  |  |  |  |  |
| 3W |  |  |  |  |  |
| 3.6W |  |  |  |  |  |
